# Observational Evidence of Large Contribution from Primary Sources for Carbon Monoxide in the South Asian Outflow

**DOI:** 10.1021/acs.est.1c05486

**Published:** 2021-12-16

**Authors:** Sanjeev Dasari, August Andersson, Maria E. Popa, Thomas Röckmann, Henry Holmstrand, Krishnakant Budhavant, Örjan Gustafsson

**Affiliations:** † Department of Environmental Science, and the Bolin Centre for Climate Research, Stockholm University, Stockholm 10691, Sweden; ‡ Institute for Marine and Atmospheric Research Utrecht (IMAU), Utrecht University, Utrecht 3584CC, The Netherlands; § Maldives Climate Observatory at Hanimaadhoo (MCOH), Maldives Meteorological Services, Hanimaadhoo 02020, Republic of the Maldives; ∥ Centre for Atmospheric and Oceanic Sciences and Divecha Centre for Climate Change, Indian Institute of Sciences (IISC), Bangalore 560012, India

**Keywords:** air pollution, incomplete combustion, atmospheric chemistry, isotopes, source apportionment, model−observation reconciliation

## Abstract

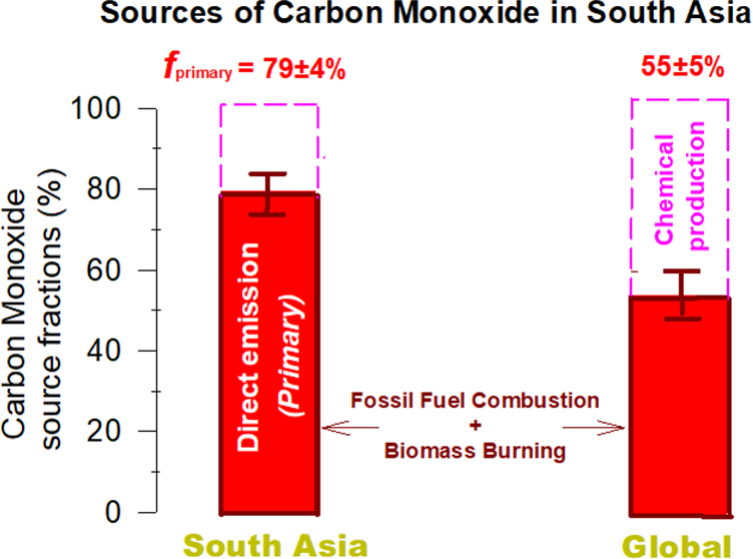

South Asian air is among the most polluted in the world, causing premature death of millions and asserting a strong perturbation of the regional climate. A central component is carbon monoxide (CO), which is a key modulator of the oxidizing capacity of the atmosphere and a potent indirect greenhouse gas. While CO concentrations are declining elsewhere, South Asia exhibits an increasing trend for unresolved reasons. In this paper, we use dual-isotope (δ^13^C and δ^18^O) fingerprinting of CO intercepted in the South Asian outflow to constrain the relative contributions from primary and secondary CO sources. Results show that combustion-derived primary sources dominate the wintertime continental CO fingerprint (*f*_primary_ ∼ 79 ± 4%), significantly higher than the global estimate (*f*_primary_ ∼ 55 ± 5%). Satellite-based inventory estimates match isotope-constrained *f*_primary_-CO, suggesting observational convergence in source characterization and a prospect for model–observation reconciliation. This “ground-truthing” emphasizes the pressing need to mitigate incomplete combustion activities for climate/air quality benefits in South Asia.

## Abstract 2

Mitigation of incomplete-combustion activities in South Asia would curtail the rising regional carbon monoxide levels benefiting air quality/human health.

## Introduction

Carbon monoxide (CO), a ubiquitous and reactive trace gas, plays a fundamental role in the global atmospheric chemistry.^1^ The main sink of CO (∼90%) is the reaction with the tropospheric hydroxyl radical (OH•).^1,2^ This reaction is reciprocally also the single largest mechanism for OH• loss (∼40%), thereby making CO an important controller of the oxidizing potential of the troposphere.^1,2^ In addition, the CO+OH• reaction plays an important role in air pollution by, for example, modulating the levels of tropospheric O_3_ (in the presence of NO_*x*_) and secondary aerosols, and is a source of atmospheric CO_2_.^3−6^ In this capacity and through chemical effects on the concentrations of other species oxidized by OH• such as CH_4_, nonmethane hydrocarbons (NMHCs), and many (stratospheric) ozone-depleting substances, CO affects the climate with an indirect positive radiative forcing estimated to be +0.23 ± 0.05 Wm^–2^ (comparable to N_2_O: +0.17 ± 0.03 Wm^–2^).^3^ Taken together, changes in CO levels have multiple implications for pollution-driven haze events, tropospheric, and even lower stratospheric chemistry, as well as trace gas budgets.^7,8^ A region currently subject to such CO-driven impacts is South Asia, where the reported trends in CO column, while being associated with some discrepancy, in general do not mirror the global decline during recent decades.^8−11^ A key knowledge gap concerns the South Asian regional sources of CO, which remain sparsely investigated and poorly understood.^10−14^

CO is emitted directly from anthropogenic combustion of fossil fuel and biomass, and produced in the atmosphere from, for example, oxidation of CH_4_ and NMHCs, and from natural biomass burning. The anthropogenic sources are at present believed to constitute about half of the total CO budget globally.^3,12,15^ However, this information from emission inventories (EIs)—an input in models used for developing the CO budget—is associated with several uncertainties.^15^ While direct comparison of the existing EI-based global CO budgets is difficult due to use of different base years, large discrepancies are found between different data sets.^15^ A key source of discrepancies is the variation in relative source contributions of CO^12,15^ (SI Figure S1). This is in part related to the large uncertainties in individual source estimates,^12,15^ NMHC oxidation (±100%), biomass burning (±50%), fossil fuel combustion (±20%), and CH_4_ oxidation (±15%). Moreover, regional variations in annual emissions of CO are often much higher for the biomass burning source (varying by a factor of 2–5).^16^ Partly related to these EI-based uncertainty issues, modeling efforts have so far had limited success in reproducing the observed strong seasonal cycle of ground-level CO concentrations in South Asia.^12−14^ As EIs form the basis for modeling of climate and air pollution effects, as well as often serve as the principal input for policy development and mitigation efforts,^3^ it is important to robustly constrain the various source fractions of CO. This will reduce the uncertainties in modeling of the climate and radiative impact in the South Asian region, the urgency being exacerbated by the increasing trend in CO column in this large region.^9^

The formation pathways of CO (direct emissions vs atmospheric chemical production) can be used to delineate source categories. Other than incomplete combustion, some forms of direct emissions of CO, such as from photodegradation or photo-oxidation of cellular material (referred as biogenic CO) and photochemical oxidation of dissolved organic matter in marine environment (referred to as oceanic CO), are known;^17−21^ however, they are reported to have a small contribution (<5%) in the global CO budget^15^ and the CO loadings over South Asia in general during winter, respectivly.^13^ In particular, during December 2017, that is, period of the winter campaign, the oceanic-CO was less than 0.4% of the total emissions flux for the geographical box covering South Asia (latitude: −4.7 to 44.5 and longitude: 63.8 to 93.8).^9^ Hence, the origin of atmospheric CO can be grouped into two main categories: primary-CO (i.e., from incomplete combustion) and secondary-CO (i.e., from secondary reactions in the atmosphere). Such a partitioning of these source classes has not yet been attempted for CO in South Asia. Furthermore, the existing estimates of CO source contributions in the region reflects a lack of consensus.^13,22−25^ The uncertainties in source attribution for this region remain large and can be ascribed to (i) difficulty to account for multiple colocated sources,^12,25^ (ii) assumptions regarding emission factors, (iii) poor a priori knowledge of source-partitioning and yield of CO during secondary production in the tropics,^19,22^ and (iv) paucity of ground-based observations making it difficult to test and constrain model simulations.^21,26^

Field-based observational constraints of CO source fractions have the potential to alleviate the knowledge deficit for the region. To this end, isotope analysis provides information that is particularly powerful for deconvolution of sources and atmospheric processess.^27−30^ The isotopic signatures enable distinguishing the primary and secondary origins of CO, as their respective sources have distinct isotope fingerprints^1^ (Supporting Information (SI) Table S1 and Notes S1–S3). The CO in an air sample can ideally be apportioned to its major sources using a combination of isotope ratios.^27^ We therefore conducted dual-isotopic fingerprinting (δ^13^C and δ^18^O) of ambient CO in the South Asian outflow sampled at a large-footprint receptor site, located on a small island, in the North Indian Ocean. Combining the outflow isotopic signatures and source endmember isotopic composition (from a newly developed isotope endmember database, compiled in the SI Excel file) within a Bayesian statistical framework enabled resolving the origin of atmospheric CO, in one of the most polluted regions in the world, during the high loading winter period.

## Materials and Methods

### Air Sampling

**Figure 1:**
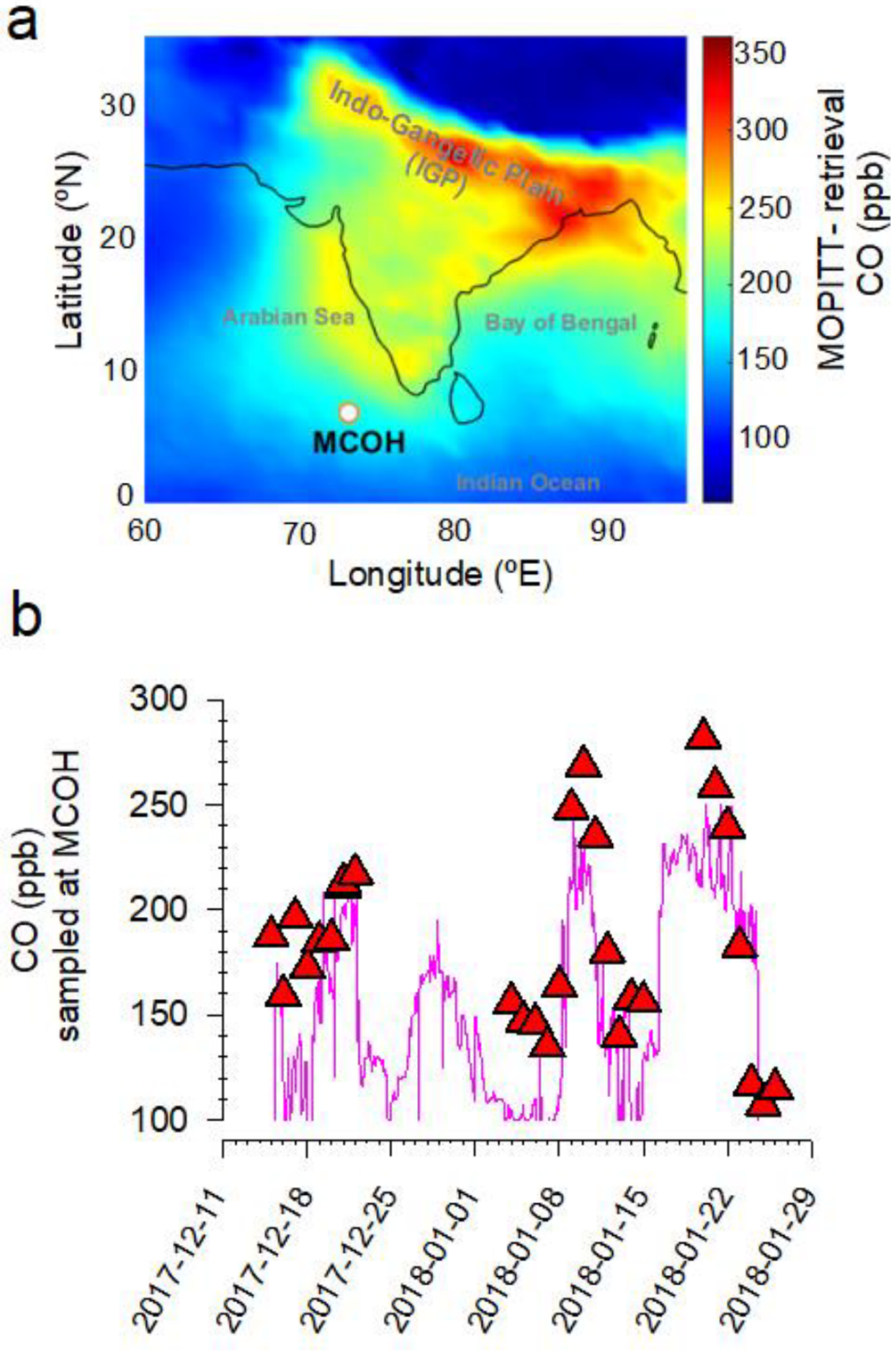
Mixing ratios of CO during December 2017 to February 2018 over the South Asian region and the sampling site Maldives Climate Observatory at Hanimaadhoo (MCOH), Republic of Maldives. (A) Multispectral CO surface mixing ratios for this period are obtained from the measurement of pollution in the troposphere instrument (MOPITT; MOP03JM v008) on board NASA’s TERRA satellite (https://terra.nasa.gov/data/mopitt-data). (B) Temporal variability of flask sampling-based CO (red triangle) and HORIBA APMA-370 instrument measured CO (pink line).

Sampling was conducted between December 2017 and February 2018 at the Maldives Climate Observatory at Hanimaadhoo (MCOH; 6.78°N; 73.18°E, 1.5m agl), located on a northern island of the northernmost atoll in the Republic of Maldives^33^ (see Figure 1 for site location). Glass flasks (1 L) equipped with polychlorotrifluoroethylene seals (Normag, Germany) were used for air sampling. They were covered with opaque rubber to block light. Prior to use, the flasks were conditioned by heating at 50 °C under vacuum (continuous evacuation) followed by flushing with nitrogen and final evacuation for a total of 5 h.^31^

Ambient air for flask sampling was subsampled from the main air inlet equipped with a PM_10_ head, connected to a 15 m tall sampling/observational tower. The air was dried using stainless steel traps (20 cm long, 10 mm OD) filled with anhydrous magnesium perchlorate (CAS# 10034–81–8, Alfa Aesar), which was changed prior to each sample collection. The drying agent was held in place by glass wool on both ends. Following drying, the air was passed through a 2 μm stainless steel particulate filter (Swagelok, Salon, OH). A portable flask sampler (built at IMAU, Utrecht University), comprising of ^1^/_4_ in. Dekabon tubing and a microdiaphragm pump (KNF Neuberger N86), was used to fill two flasks in series with this dried air compressed to an absolute pressure of ∼1.7 bar. Prior to isolating the air sample, the glass flasks were flushed for 20 min, at a flow rate of 2 L min^–1^. Overall, in this study, 56 samples (i.e., 28 batches) and four blanks were collected (details in SI Table S2).

The ambient CO concentration at MCOH is continuously measured using a nondispersive infrared CO analyzer (HORIBA APMA-370) (see SI Figure S3). The instrument was calibrated using a 1 ppm of CO standard (Nippon Gases Sverige AB) in the beginning of the flask sampling campaign. Calibration was checked once a week during the campaign. The quality control was carried out using a 100 ppb CO standard (Nippon Gases Sverige AB).

### Extraction of CO and Measurement of Stable Isotopic Composition

The CO mixing ratio and isotope measurements were carried out at IMAU (Utrecht University) using a continuous-flow isotope ratio mass spectrometry (IRMS) method.^32^ The standard measurement procedure is detailed elsewhere^32^ and only briefly summarized here: sampled air from glass flasks is injected into an extraction system. First, CO_2_ and N_2_O are removed from the air matrix using an Ascarite/Mg(ClO_4_)_2_ and a liquid nitrogen trap. Subsequently, CO is selectively oxidized to CO_2_ on Schütze reagent (sulfuric acid combined with I_2_O_5_ on granular silica gel). CO-derived CO_2_ is then separated from O_2_ and N_2_ cryogenically, further purified with a gas chromatographic column, dried via a Nafion dryer, and then transferred via an open slit interface^33^ to the IRMS (Thermo Scientific Delta V Advantage) for isotopic analysis. The final CO isotope values are calculated from the measured CO_2_ isotope values, correcting for the extra oxygen atom transferred from the Schütze reagent. Mixing ratios are calculated based on peak area and reported in parts per billion (ppb).

Isotope values are reported in δ notation, as the relative deviation of the ratio of the minor isotope to the abundant isotope in a sample (SA) relative to an international standard (ST), that is, δ^13^C = (^13^R_SA_/^13^R_ST_ – 1) × 1000‰. In the case of δ^13^C, the standard is Vienna Pee Dee Belemnite (V-PDB), in the case of the δ^18^O it is Vienna Standard Mean Ocean Water (V-SMOW). The mixing ratios and isotope values were calibrated using a reference cylinder filled with atmospheric air with known mixing ratio and isotopic composition^31,32^ (NAT-307; CO = 180 ppb; δ^13^C = −30.25‰; δ^18^O = +7.10‰). The accuracy and repeatability of the system were determined by analyzing cylinders with known composition.^32^ The analytical precision (1-sigma repeatability) for the measurements reported here was estimated at 0.12‰ for δ^13^C and 0.16‰ for δ^18^O.

## Results and Discussion

### Wintertime CO Mixing Ratios in South Asia

Elevated levels of aerosols and trace gases is a characteristic of wintertime South Asia^25,34^ (Figure 1a). The vast regional emissions combine with a shallow boundary layer during the NE monsoon system to result in massive buildup of pollutants across North India with subsequent dispersal out over the Bay of Bengal and the North Indian Ocean.^35^ The high but uneven CO loadings across the continent, therefore, reflect the combined roles of seasonal meteorology, anthropogenic activities, distribution of CO sources, and atmospheric chemistry.^10,22−25,34^ CO in this region has a lifetime of a few weeks during winter (see SI Figure S2), which is both short enough for CO to exhibit spatial heterogeneity yet long enough to still be influenced by synoptic-scale regional air mass transport.^34,35^ Hence, sampling at the Maldives Climate Observatory at Hanimaadhoo (MCOH; Figure 1a), located downwind of the continent during winter, allows to intercept and fingerprint the sources as well as trace the geographical emission regions contributing to the CO transported from the continent to the North Indian Ocean.^36^

Changes in source characteristics, atmospheric transport pathways, and sinks influence the CO mixing ratios.^37−39^ Our flask sampling-based CO mixing ratios ranged from 108 to 282 ppb—showing high variability on the time scale of one to a few days—during the winter period and compared well with continuous online CO measurements, as well as aerosol number concentrations and loadings of black carbon aerosols, similarly stemming from common sources upwind in the continent (Figure 1b; see also SI Figure S3). The high short-term variability of CO at MCOH is in contrast to observations from remote sites in other locations.^37,38^ The strong variability cannot be caused by variations in the sink, because the [OH•] variability is not large and/or fast enough^2,40^ to contribute significantly to the observed CO variability on the short time scales characteristic of this regional system. The most plausible explanation for the observed variability must then be changes in the source regimes coupled to shifting air masses.

Analysis of air mass trajectories confirms that the variability of CO mixing ratios at MCOH is indeed related to the extent of influence from the continental outflow. During transition periods (e.g., 14–16, 23–25 January, respectively), wherein air masses shifted from predominantly continental to background-marine regime, the mixing ratios were reduced, by as much as 150 ppb, to levels characteristic of remote regions^20,37,38^ (SI Figures S4–S5). The highest mixing ratios were associated with air arriving from the Indo Gangetic Plain (IGP) - Bay of Bengal transport sector (enhancements of 180 ppb and above), highlighting the intensity of emissions from the densely populated and highly polluted IGP region^39^ (SI Figure S4). The widely varying air mass back trajectories and the corresponding changes in CO also illustrate that the sampling site is not dominated by local emissions and is representative of a wider regional South Asian footprint.^36^ To extend our analysis beyond the determination of geographical source origins, we used stable isotopes to deconvolute the emission sources of CO.

### Isotope Signals of CO Intercepted Over the North Indian Ocean

**Figure 2:**
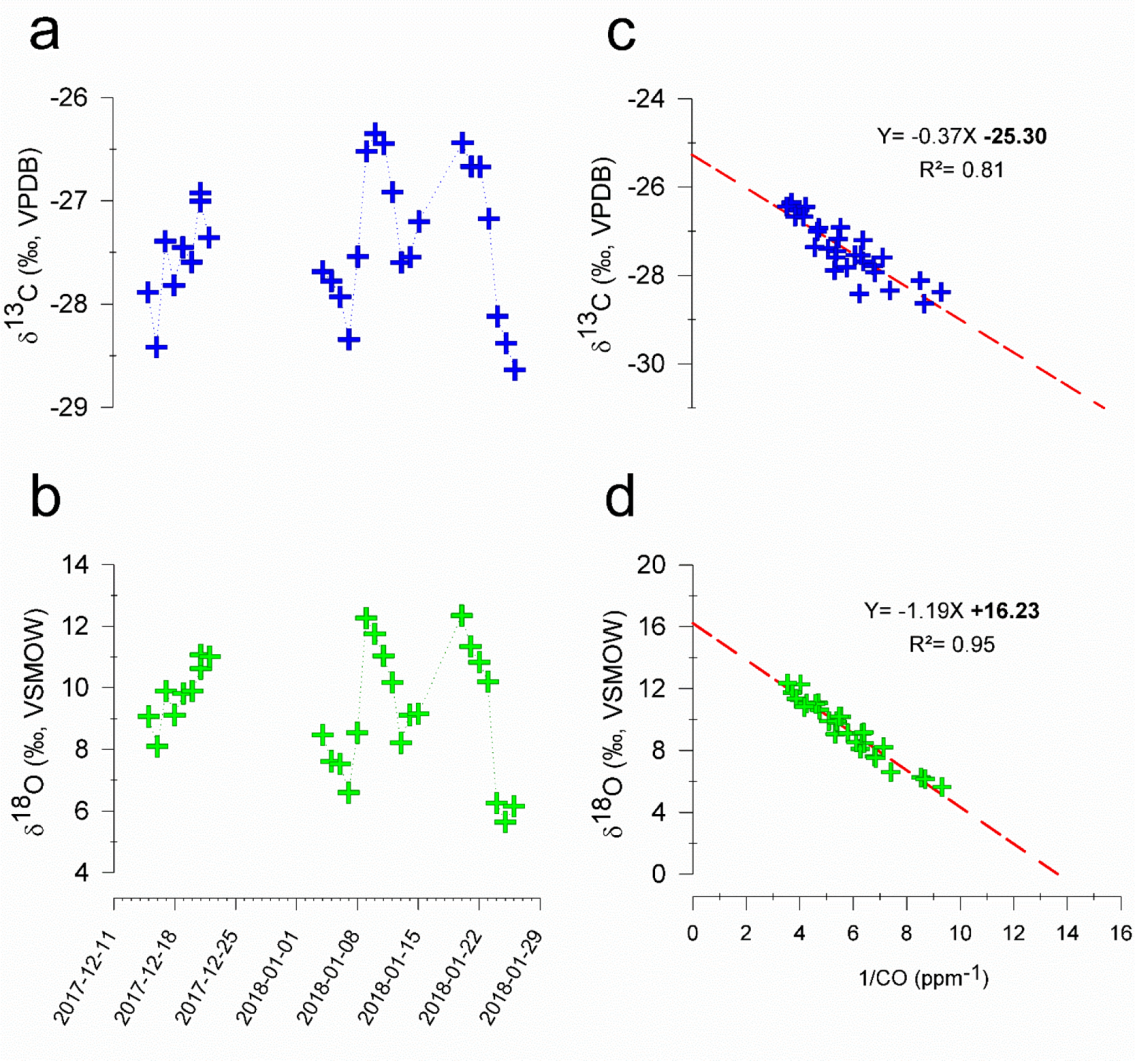
(A, B) Temporal evolution of carbon (δ^13^C; blue) and oxygen (δ^18^O; green) isotope ratios of CO. (C, D) Keeling-plots for δ^13^C and δ^18^O with linear regressions and corresponding correlation coefficients is shown for both sites. The coevolution of CO and Black carbon (BC) concentrations during the winter campaign of 2018 is shown in SI Figure S3a.

**Figure 3:**
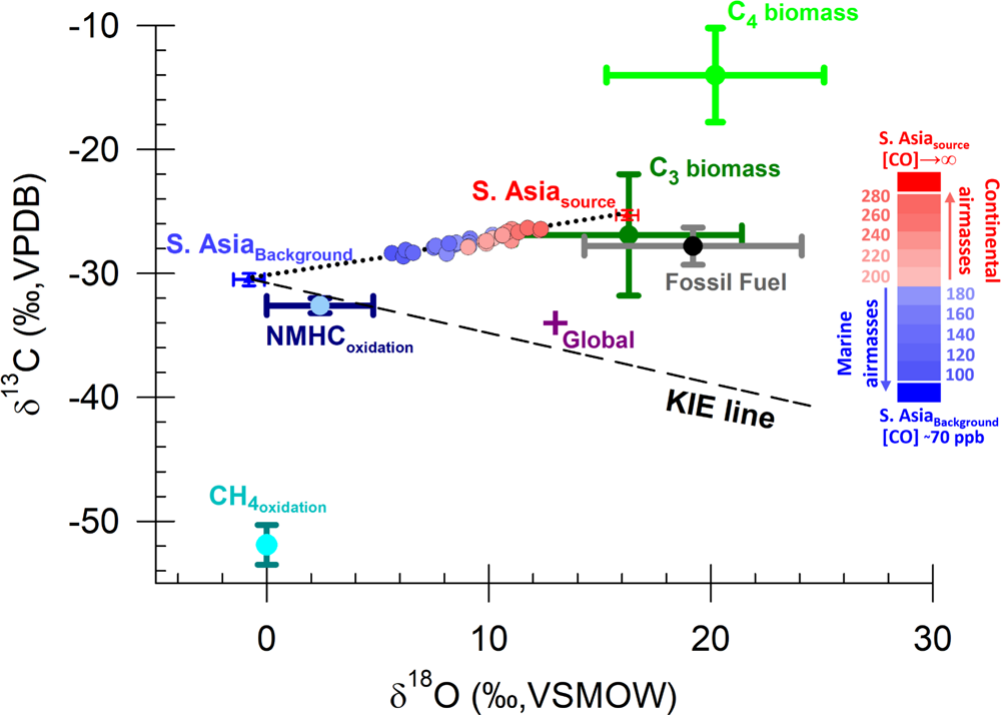
Observed CO isotopic signatures at MCOH site (circles filled) and predicted source-mixing line (MCMC fit in black dotted line; see also SI Note S7) fall on a tight line connecting the regional background (blue; South Asia_background_; see also SI Notes S5–S6 and Figure S8) and continental source (red; South Asia_source_). The sources of CO and corresponding isotopic signatures are shown for biomass burning (C3 plants, C4 plants), fossil fuel combustion, oxidation of NMHC, and CH_4_, respectively (see SI Tables S1 and S3 and SI Excel file for endmember compilation from previous studies). The global average CO isotopic signature (purple)^3^ is also shown. The potential effect of the sink reaction with OH on the background signal (kinetic isotope effect (KIE) line)^30^ is investigated using a theoretical model (see SI Note S6). The blue-to-red color bar represents the CO concentrations corresponding to the airmasses at MCOH during the winter campaign. Lower CO mixing ratios suggest a larger influence from background, and consequently higher CO mixing ratios suggest larger influence from the strong continental sources upwind (see also Figure 1).

At MCOH, the stable isotopic composition (δ^13^C and δ^18^O) of CO showed changes concomitant with the CO mixing ratios (Figure 2). The arrival of continental polluted air masses was accompanied by increases in both δ^13^C and δ^18^O. A significant variability was observed in δ^13^C (∼3‰), while the continuous and gradual enrichment in δ^18^O was even more pronounced (∼7‰). The maxima in the observed δ^13^C and δ^18^O signals were close to the isotopic signatures of CO produced from fossil fuel combustion and C3 biomass burning (SI Table S1; see also Figure 3). In addition, mixture of CO from C4 biomass burning with the CO pool downwind of the continent can also lead to the observed maxima of δ^13^C and δ^18^O of CO sampled at MCOH.

Indeed, the wintertime sensitivity of emissions reaching MCOH is found to be a combination of emissions from the IGP and peninsular India (south of <23.4°N).^36^ Crop-residue burning of sugar cane and associated bagasse-based power generation is more widespread in peninsular India than in the IGP during winter.^36,41^ Such C4 plant burning sources emit ^13^C-enriched CO, which likely contribute to the observed enrichment of the isotopic signals sampled downwind over North Indian Ocean.^42,43^ Hence, in addition to fossil fuel combustion and C3 biomass burning, C4 biomass burning is a plausible primary source in the context of South Asian continental emissions of CO, as has also been recently shown for incomplete combustion-derived black carbon aerosols investigated at the same site.^36^ Taken together, based on the observed sharp positive trend in δ^13^C and δ^18^O during polluted air mass regime, we hypothesize that formation of CO is likely driven by a combination of primary sources. For further source-diagnostics the source signatures representative of the dominant air mass regimes is investigated.

### Fingerprinting the Isotopic Signature of the Continental Source

For a quantitative apportionment of the sources, including the effect of the admixture of background air, the Keeling-plot approach—drawing upon a linear relation between isotope signatures and the inverse of concentrations—is often useful^28,44^ (details in SI Note S4). Here, we observe high and significant linear correlation coefficients for both δ^13^C and δ^18^O relative to [CO]^−1^ (*R*^2^ = 0.81 and *R*^2^ = 0.95, respectively; *P* < 0.05), which suggests that the underlying assumption of the Keeling-plot approach are reasonably met in this wintertime South Asian system (see Figure 2). This implies that the source profile of CO in the South Asian outflow intercepted at MCOH can be modeled as a two-component mixture, where a background signal is modulated by a temporally varying strong source signal.

The *y*-axis (δ^13^C or δ^18^O) intercept of the mixing line in the Keeling plot corresponds to the source signature. Such an analysis of the current data set revealed the C and O isotope signatures of this source, δ^13^C = −25.3 ± 0.4‰ and δ^18^O = +16.2 ± 0.5‰, to be clearly combustion driven (Figure 2c,d). Given that the high concentration regime in this data is driven by air masses originating from the South Asian outflow, the isotope signatures together represent the source fingerprint of continental CO (Figure 3). The South Asia_source_ has an isotopic composition overlapping with the primary CO sources (Figure 3; see also SI Table S1), corroborating the arguments in the previous section. This implies that the atmospheric CO in the South Asian outflow intercepted at MCOH is driven by the mixture of primary CO from mixed pollution sources in the continent with a relatively constant isotopic composition of CO in the background air. The observation that the slope of the dual isotope correlation of the data line has a different sign compared to what would be expected from KIE further reinforces our interpretation that this trend is due to source mixing, rather than atmospheric sink reactions (Figure 3).

Secondary CO sources such as oxidation of NMHC and CH_4_ can also contribute to the overall isotopic signal.^44,45^ Biogenic NMHCs (e.g., isoprene) are reported to be the globally largest component of the NMHC oxidation source of CO.^19−21^ Recent modeling estimates suggest a low biogenic NMHC-derived CO flux in South Asia compared to other tropical regions such as central Africa and parts of S America.^21^ Furthermore, photosynthesis driven emissions of biogenic NMHCs are stronger during summer,^19,44^ implying that this source should have only a relatively small contribution to the isotopic signature of continental CO in wintertime.^13^ However, the anthropogenic NMHCs (such as ethane) can get oxidized to CO on short time scales and can contribute to the overall CO isotopic signal. While the contribution of anthropogenic NMHCs-derived CO remains unclear due to poor knowledge of yields and emission information specially in the tropics,^12,19^ a small contribution from this source could have a substantial effect on the overall isotopic signature of CO. This is because anthropogenic NMHC-derived CO carries a near-zero δ^18^O, upon oxidation, the isotopic composition of this CO would get further depleted in ^18^O^44,45^ due to the kinetic isotope effect (KIE) in reaction with OH•.^30^ Therefore, even a small contribution from this source would draw the South Asia_source_ signal away from the primary “source triangle” (see Figure 3). Together, this further strengthens our arguments above that primary sources could indeed have a large contribution to the CO isotopic signal. Due to the long lifetime of CH_4_ of about 9 years,^34^ this source does not contribute strongly to plume signals but represents a large fraction of the background endmember, the marine air mass influenced periods at MCOH. Therefore, in the present context, secondary CO in the South Asian outflow could mainly be CO from anthropogenic NMHC oxidation.

Given the two-member mixing model, the isotopic signature of the background CO can also be derived from the Keeling plot. By establishing the lowest CO mixing ratio encountered at MCOH during the background-marine air mass regime (SI Notes S5–S6; see also SI Figures S3 and S8), we deduced the corresponding isotopic signatures using the mixing-line equation in the Keeling plot (at [CO] ∼70 ppb; δ^13^C = −30.5 ± 0.5‰; δ^18^O = −0.8 ± 0.7‰). The background CO isotopic signature at MCOH is similar to the ones reported from other remote regions.^20^ By combining the above information, we quantitatively constrained the primary and secondary source contributions of CO over South Asia.

### Source Quantification Based on Statistical Modeling

The isotopic fingerprint of the South Asia_source_ signal overlaps with the signatures of primary sources of CO (Figure 3; see also SI Table S1 and SI Excel file). By combining the two stable isotopes, the relative contributions of these sources can be constrained further.^27^ However, the direct quantification approach faces two overall complications: (i) isotope signatures of the fossil fuel combustion and C3 biomass burning endmembers are largely overlapping, (ii) there are three potential primary sources (C3 biomass burning, C4 biomass burning, fossil fuel combustion) and one secondary source (NMHC-oxidation; see arguments above) which can contribute to the South Asia_source_ signal; separation of four components using two markers leads to an under-determined system of equations. Although an under-determined system may still be solved using a simplified Bayesian statistical model,^36,43^ the associated uncertainties generally become large, and source-separation becomes ill-determined.

**Figure 4:**
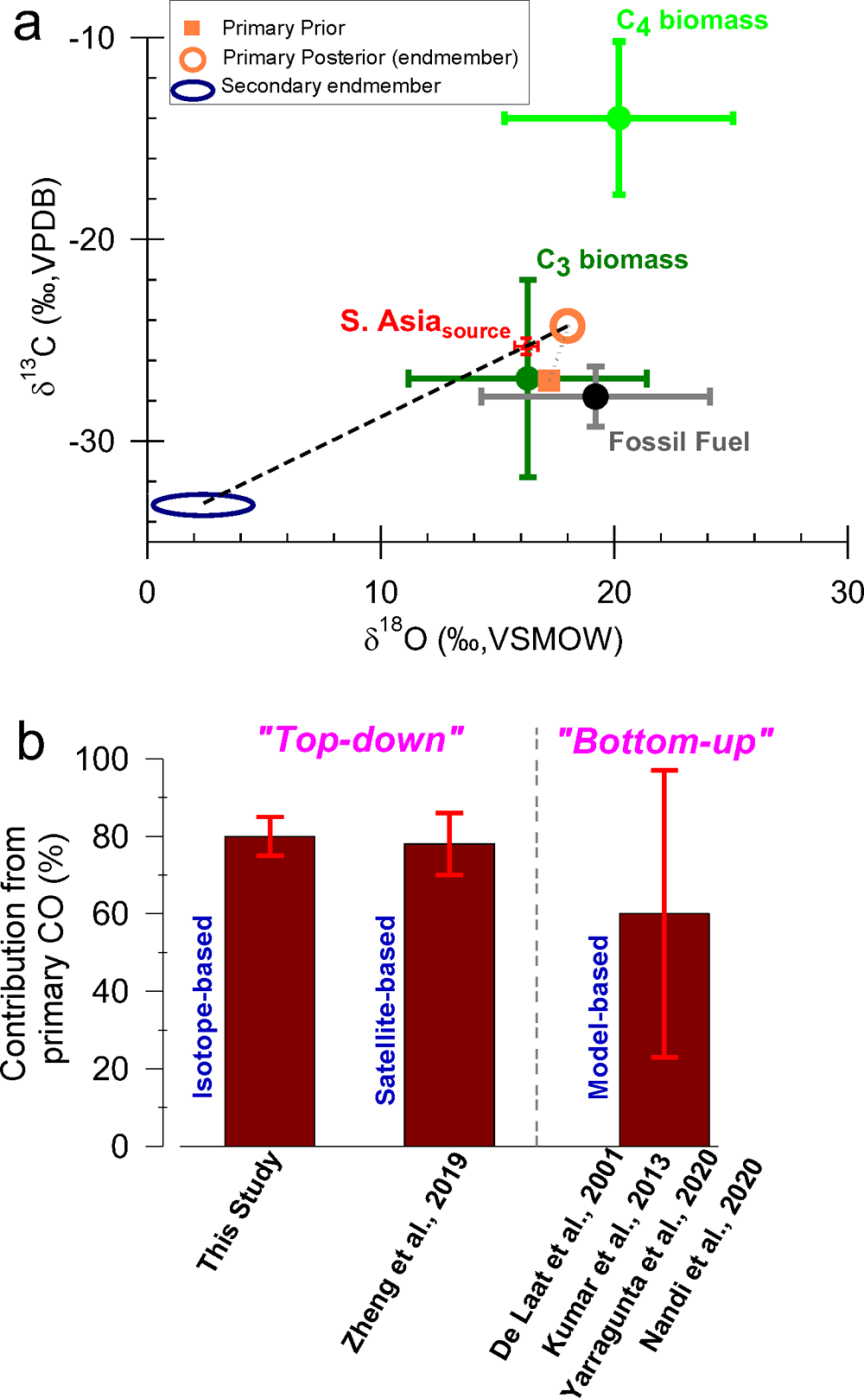
South Asian_source_ signal (as in Figure 3) is apportioned using endmembers for primary CO and secondary CO (mathematical formulation in SI Note S7). (A) The dashed line (black) represents mixing between primary and secondary CO. The primary endmember (orange, open circle) is deduced from two scenarios–informed prior (orange, square; where the contribution of the three primary sources is estimated from bottom-up emission inventories; details in SI Note S8), and uninformed prior (shown in SI Figure S9; where any source contributions is assumed to be equal). The secondary CO endmember (blue, ellipse) is the same as the NMHC_oxidation_ source (see Figure 3 and arguments in discussion section; see also SI Table S1). (B) The fractional contribution of primary CO (*f*_primary_) computed using the isotope-based hierarchical Bayesian model is shown (see also SI Figure S6). The isotope-based source fractions of CO are compared with a satellite-derived estimate^9^ and regional modeling-based estimates^13,23,46,47^ (see also SI Figure S1).

To quantitatively constrain the fractional contributions of primary- (*f*_primary_) vs secondary- (*f*_secondary_) CO in the South Asia_source_ signal, a hierarchical Bayesian statistical model was developed (see details in SI Note S7). As anthropogenic NMHC oxidation-derived CO can be produced on shorter time scales^19^ similar to that of wintertime CO lifetime in South Asia (see SI Figure S2), the isotopic endmember for the secondary CO was kept the same as that of the NMHC-oxidation source (see SI Table S1). The isotopic endmember of the primary CO is a mixture of three different sources (C3 biomass burning, C4 biomass burning, fossil fuel combustion). However, the relative contributions are uncertain. To address this issue, two scenarios were explored (i) with an informed Bayesian prior: the relative contributions of the three sources are based on a combination of estimates from two bottom-up emission inventories of CO^47,48^ (65% C3 biomass burning; 5% C4 biomass burning; 30% fossil fuel combustion; see SI Note S8); (ii) with an uninformed (flat) prior: equal contribution from the three sources. The two scenarios were then used as a priori in the Bayesian framework and a Markov chain Monte Carlo (MCMC) approach was used to estimate the posteriors (Figure 4a).

The resulting fraction of the primary source, *f*_primary_ is remarkably similar for both scenarios: 79 ± 4% (with informed prior) and 76 ± 4% (with flat prior) (Figure 4b; see also SI Figures S6 and S9). As such, based on these statistical modeling scenarios, we conclude that South Asian wintertime continental emissions of CO have a dominant contribution from primary sources (Figure 4b). Furthermore, the posterior distributions for the two scenarios are overlapping (see SI Figure S6), demonstrating that the simulation is not sensitive to the chosen priors, and that the system provides a robust constraint for the primary endmember variability. Thus, even though the separation of the individual primary sources is complex, we conclude that the estimation of the total primary CO contribution is rather insensitive to the primary endmember configurations.

### Model vs Observation Comparison

The CO concentrations in South Asia have been rising in the past decades.^9,10^ This region is now contributing ∼20% to the global CO budget.^9^ While South Asia is currently experiencing rapid economic/population growth, with associated severe wintertime air pollution, the specific drivers of the positive CO trend remain unclear. Using isotope-based diagnostics, we find CO is largely driven by primary sources in this region (*f*_primary_ 79 ± 4%). This *f*_primary_ for South Asian continental CO (South Asian_source_ in Figure 3) is significantly higher than estimated for global CO (55 ± 5%),^3^ emphasizing the regional-specific source regime.

So far, only a limited number of modeling studies have focused on source apportionment of CO in South Asia, despite the growing need to identify potential drivers of its increasing trend and the CO-induced air pollution.^13,23,49,50^ These existing studies rely on tracers as well as on the tagged emission approach for investigating both sources and source regions contributing to surface CO during winter. Overall, the different models find the *f*_primary_ to be poorly constrained , with estimates spanning between 34% and 97% during winter, likely reflecting the implementation of different chemical transport models coupled to different bottom-up emission inventories, which in turn are also associated with large uncertainties^13,23,49−51^ (Figure 4b). The model–observation mismatch in the atmospheric abundance of CO in the South Asian region is a long-standing and unresolved issue.^49^ An overall offset between modeled concentrations and observations also translates into uncertainty in the estimation of the atmospheric abundance of key atmospheric oxidants such as OH and O_3_, with implications for air pollution, atmospheric chemistry, and regional climate. The specific causes for these offsets are not well constrained, potentially reflecting issues with both emission input and/or model performance. Source-resolved observational data reported here (*f*_primary_ 79 ± 4%) is expected to offer a way forward toward resolving these issues.^36^ Meanwhile, this information is also of direct relevance for policy developments, seeking to minimize the severe air pollution situation in the region.

### Deconvolution of the Model–Observation Mismatch

Regarding the emission estimates, comparison of different emission inventory-coupled models shows that estimates of *f*_primary_ vary by as much as a factor of ∼3 (Figure 4b). A major source of uncertainty in emission inventories is likely associated with biofuels (fuelwood, charcoal, dung cakes, agricultural residue).^12^ As the sales of such fuels are localized with no nationalized inventorying, the activity levels and emission factors remain a source of high uncertainty. Global inversion analyses of CO have found that emission inventories, used in the CO modeling studies for South Asia, significantly underestimate Asian emissions.^12^ In most cases where emission factors for CO from South Asia are unavailable, the emission factors are assumed to be identical as for use of similar fuels in developed countries. However, emissions factors are likely different in different regions with different fuel condition and combustion systems.^52^ In fact, certain regional sources such as brick kilns, diesel generators, kerosene usage, production of coke, iron, and steel when accounted are believed to have led to most of the increase in emissions in regional East Asian inventories.^12^ These are unfortunately still “missing” in many emission inventories for the South Asian region.^48^

Regarding model performance, the CO-background contributions (*f*_background_) apportioned in the modeling studies is likely a contributing factor for the mismatch with observations.^23,49^ The unresolved fraction of CO in modeled results using domain boundaries is often attributed as CO-background.^13,23,49^ However, issues with parametrization of regional-scale transport within domain boundaries in global models has been reported for the South Asian region.^53^ Generally, anthropogenic CO over South. Asia is found to be largely from surface emissions in the region with little influence from neighboring regions such as Southeast Asia and Africa, even though the modeled background CO is occasionally found to be as high as 60% on average and as high as 74% during winter.^13,23,49,50^ These estimates can be compared with results from the present study. Using an isotopic mass-balance apportionment with the presently constrained δ^18^O endmember background (−0.8 ± 0.7‰) and South. Asian source (+16.2 ± 0.5‰), we find that the *f*_background_ ranges between 56% (during marine air mass origins) and 23% (during IGP air mass origins) at MCOH. Intuitively, this implies that *f*_background_ CO in mainland South Asia should be substantially lower (<23%) than reported by modeling studies.^13,23^ Thus, direct comparison with observational constraints suggests that *f*_background_ CO is indeed overestimated in models and likely also contributing to the model–observation mismatch. Taken together, the uncertainties in emission inventories lead to a poorly constrained *f*_primary_ CO, and issues with parametrization of domain boundaries in models possibly lead to overestimated *f*_background_ CO.

The isotope-based observational constraints on CO source fractions can also be compared with other “top-down” observational estimates. In general, satellite-based inversion approaches have been the norm for studying long-term CO trends and understanding the global CO budget^9,29,54−56^ (see also SI Figure S1). One such satellite product—Measurement of Pollution in the Troposphere (MOPITT)—has been widely used for this purpose.^54−56^ However, substantial uncertainty exists depending on the type of inversion and other assimilations used in the process of deconvoluting the CO sources.^54−56^ Furthermore, sensitivity issues exist for lower tropospheric vs column (and profile) retrievals using MOPITT.^56^ Recent evidence suggests underestimation of observed CO concentrations by MOPITT despite a good match with modeled CO concentrations.^53^ Such issues call for the validation of the satellite-derived estimates of CO source fractions, that is, for “ground truthing” using observations.^7−10,21,24,54−56^ Recently, by coupling multiple satellite products within a Bayesian model, source-resolved emission fluxes were computed,^9^ including contributions from both primary and secondary emissions. This top-down estimate for the fraction primary for South Asia (79%) for the wintertime period (December 2017) overlaps with the observation-constrained *f*_primary_ in the present study (79 ± 4%; Figure 4b). Thus, we here see convergence between two different, and complementary observational approaches. The isotope-based results, thereby, provide much-asked for validation of the satellite-based estimates. Furthermore, these results suggest that the present study provides a good test bed for testing “bottom-up” based modeling studies for CO in South Asia against source-resolved data. It also provides the opportunity of isolating model skill from uncertain emission estimates when comparing atmospheric transport model results with observations data, by coupling to the well constrained and presently validated top-down emissions inventory.

Overall, the findings in this work emphasize the importance of incomplete combustion for CO emissions from winter-time South Asia. As such, this has clear implications for society: primary emissions dominate the CO that aggravates regional air pollution, while also contributing to regional warming. Intuitively, a similar contribution from *f*_primary_-CO on a year-round scale would imply that a significant reduction of CO from such incomplete combustion emissions in South Asia could result in substantial feedbacks affecting, for example, the lifetime and climate warming of atmospheric CH_4_ owing to the CO–OH–CH_4_ chemical coupling.^8^ Meanwhile, the total effect from mitigating incomplete combustion in South Asia, including the broad cocktail of coemitted substances such as organic and black carbon aerosols, NO_*x*_ and SO_2_ would reduce air pollution even further.^57^ A year-round observational study of CO source dynamics in South Asia is therefore warranted to better understand and model these effects.

Taken together, the present study provides observation-based scientific underpinning of the possible drivers of the recent decadal South Asian CO trend and facilitates the development of targeted mitigation measures, as well as providing improved possibilities for constraining modeling of the climate and air quality/health impact of CO in one of the most polluted and climatically vulnerable regions in the world during the wintertime continental outflow.

## Supporting Information Available

Notes S1–S3 include discussions on the South Asia-specific isotope endmembers and fractionation effects in different CO sources. Notes S4–S8 include discussion on the source apportionment process employed in this study. Figures S1–S9 include back trajectories, various aerosol parameters monitored at MCOH, source apportionment results for different modeling scenarios and vehicular CO isotope fingerprints. Tables S1–S3 list the sampling details and summary of CO isotope endmembers (PDF)
Excel file with Isotope signatures (endmember values) of various sources (XLSX)
