# Supplementary material for: Observational Evidence of Large Contribution from Primary Sources for Carbon Monoxide in the South Asian Outflow
Source: Environ Sci Technol. 2021 Dec 16;56(1):165–74. doi: 10.1021/acs.est.1c05486 (PMC8733925; doi:10.1021/acs.est.1c05486)
Supplement: Supplementary file 1 — es1c05486_si_001.pdf [file es1c05486_si_001.pdf]

# Supporting Information

## Observational evidence of large contribution from primary sources for carbon monoxide in the South Asian outflow

*Sanjeev Dasari[1#], August Andersson[1*], Maria E. Popa[2], Thomas Röckmann[2], Henry Holmstrand[1], Krishnakant Budhavant[1,3,4] and Örjan Gustafsson[1]*

[1]Department of Environmental Science, and the Bolin Centre for Climate Research, Stockholm University, Stockholm 10691, Sweden

[2]Institute for Marine and Atmospheric Research Utrecht (IMAU), Utrecht University, Utrecht 3584CC, the Netherlands

[3]Maldives Climate Observatory at Hanimaadhoo (MCOH), Maldives Meteorological Services, Hanimaadhoo 02020, Republic of the Maldives

[4]Centre for Atmospheric and Oceanic Sciences and Divecha Centre for Climate Change, Indian Institute of Sciences (IISC), Bangalore 560012, India

Current address: [#]Institute Des Géosciences De L'Environnement (IGE), University Grenoble Alpes, CNRS, IRD, Grenoble INP, Grenoble 38000, France

[*]Corresponding Author: Phone: +46 816 4015; E-mail: august.andersson@aces.su.se

**Supporting information includes**: 34 pages, 8 Notes, 9 Figures, and 3 tables

# Contents

## *Supplementary Notes*

Note S1. A discussion on the isotopic fractionation of CO produced in vehicular emissions ……….………………………..S3
Note S2. South Asia-specific endmember for CO from fossil fuel combustion ……………………………………………S5
Note S3. A discussion on the isotopic fractionation of CO produced in biomass burning…………………………………… S6
Note S4. A discussion on the Keeling-plot approach ……………………………………………………………………….S8
Note S5. A discussion on the background CO levels at MCOH………………………………………………………….S9
Note S6. A theoretical model accounting for the effect of scavenging process on the background CO signal at MCOH……S10
Note S7. Source apportionment based on a hierarchical Bayesian statistical model.……..…………………………………..S12
Note S8. Establishing an informed prior using emission estimates from bottom-up inventories……………………………..S16

## *Supplementary Figures*

Figure S1. Fractional contribution of major source categories to global carbon monoxide (CO) budget……………………..S17
Figure S2. Wintertime (December to February) lifetime ($\tau$) of carbon monoxide (CO) ………………….…………………..S18
Figure S3. Online measurements of CO and aerosol parameters at MCOH…………………………………………………...S19
Figure S4. Air mass transport pathways for CO sampled at MCOH …………………………………………………………S20
Figure S5. Air mass fractional cluster contribution …………………………. ………………………..............................S22
Figure S6. MCMC-based probability density functions for the fraction primary CO ……………………………………... S23
Figure S7. Stable isotopic signatures of CO from fossil fuel combustion in vehicular emissions ………..…………………...S24
Figure S8. The trajectory of the background signal at MCOH with reference to the scavenging process (KIE line) ………..S25
Figure S9. Bayesian dual-isotope source contribution modeling of CO in South Asia...……………………………………...S26

## *Supplementary Tables*

Table S1. Dual-isotope [carbon ($\delta^{13}$C) and oxygen ($\delta^{18}$O)] endmembers for different CO sources…………………………..S27
Table S2. Flask-based sampling of carbon monoxide (CO) at the Maldives Climate Observatory at Hanimaadhoo...………S28
Table S3. A compilation of the isotopic signatures [ ($\delta^{13}$C, $\delta^{18}$O)] of carbon monoxide for different sources……………….S29

***References* (S30 – S34)**

# Supplementary Notes

## Note S1. A discussion on the isotopic fractionation of CO produced in vehicular emissions

The isotopic signatures of CO emitted from fossil fuel-based traffic emissions show are quite variable in both the $\delta^{13}C$ and $\delta^{18}O$ dimensions (see Figure S7). Central to these variations are petrol vs. diesel emissions as well as catalytic processes[1-3]. The vehicle-to-vehicle variation of emitted CO and catalytic efficiency are judged using, for instance, $CO:CO_2$ and $H_2:CO$ ratios, respectively[3]. While diesel engines have a higher combustion efficiency, thereby largely oxidizing CO to $CO_2$, petrol engines run at the stoichiometric point with just enough oxygen to burn all fuel, resulting in high CO emissions. A three-way catalytic converter (TWC) is used in petrol engines, in part, to oxidize this CO. The TWC performs sub-optimally when it is not sufficiently heated (referred to as cold start emissions) and during lack of enough $O_2$. These conditions affect the isotopic signatures of CO produced in the engine[3].

*Variations in $\delta^{13}C$*

A clear distinction is found in CO sampled from individual stationary vehicles tested by varying parameters such as engine status (e.g., idling, revving), load, and speed ($\delta^{13}C$=-26±12‰; $\delta^{18}O$=25±7‰) vs. from a fleet of moving vehicles ($\delta^{13}C$= -27±2‰; $\delta^{18}O$=19±5‰) (see Figure S7). It is noteworthy that mean $\delta^{13}C$ of a fleet in different urban locations, highways and tunnels are similar, whereas the spread in $\delta^{18}O$ is larger. The spread in $\delta^{13}C$ also gets muted to a large extent when comparing fleet with individual vehicles. High-emitting vehicles (e.g., cold petrol engines) with a large spread in $CO:CO_2$ show a gradual enrichment in $\delta^{13}C$ (relative to that of the fuel) with the oxidation of CO and therefore contribute significantly to the spread in the isotopic signatures when tested individually in stationary conditions[1,3]. Likewise, some vehicles with extremely low $CO:CO_2$ have also shown a depleted $\delta^{13}C$ relative to that of the fuel, indicating complexity of emission systems[3]. However, this effect is completely subdued in parking garage (low-speed cold-engines) vs. highway (high-speed hot-engines) comparison of a fleet of moving vehicles, implying i) the difference in driving conditions does not result in a significant difference in the integrated $^{13}CO/^{12}CO$ composition, ii) despite the different regimes, the overall isotopic signatures of CO in traffic possibly are dominated by the isotopic signatures of CO from the high-emitting vehicles, iii) the low $CO:CO_2$ scenarios (depleted $\delta^{13}C$) of vehicular emissions do not affect the overall traffic signature[3]. Since nearly all carbon leaves the vehicle as $CO_2$, for a large range of moderate $CO:CO_2$ ratios, it is reasonable to assume that the $^{13}CO$ is closer to or slightly enriched than the C-isotopic signature of the fuel (~ -30 to -26‰)[4].

*Variations in $\delta^{18}O$*

Oxygen leaves the vehicle as $H_2O$, and since $CO_2$ can undergo isotopic exchange with $H_2O$, the $\delta^{18}O$ deviates from atmospheric oxygen (23.9‰)[5]. A conspicuous observation from the comparison of individual stationery vehicular emissions and fleet emissions of CO (see Figure S7) is that diesel engines show isotopically depleted $C^{18}O$ compared to petrol engines. This could be attributed to the combustion efficiency of diesel engines. The kinetic isotope effect (KIE) in the CO+OH· reaction (i.e., destruction of CO) induces a negative enrichment of (upto ~-10‰) in $^{12}C^{18}O$, implying the residual CO will be depleted in $\delta^{18}O$[1,6]. Nonetheless the shift in $\delta^{18}O$ is still uncertain in petrol engines, wherein a positive enrichment in $^{18}O$ (normal KIE) has also been found during the destruction of CO in heavier

engines as well as negative enrichment (inverse KIE) in certain smaller engines[1,3]. However, one aspect determining the shift is the presence/absence of a catalytic converter as well as the metal surface of the catalytic converter which have both shown to cause large variations in $C^{18}O$, respectively[1,7]. Based on the observations (in Figure S7), it is found that cold diesel emissions often form a distinct isotopic cluster compared to cold petrol emissions. Taken together, the reasons for the spread in $\delta^{18}O$ are not well known and possibly related to the several factors including the engine-size, fuel-type, vehicle age[1,3,7-9]. Overall, it is reasonable to assume that the $C^{18}O$ is often slightly depleted than atmospheric $O_2$.

## Note S2. South Asia-specific endmember for CO from fossil fuel combustion

Emissions from traffic constitute a major fraction of the fossil fuel usage in South Asia[10]. The vehicular fleet, in this region, can be grouped into 2-wheelers (2-W), 3-wheelers, 4-wheelers, low duty diesel (LDDV) and high duty diesel vehicles (HDDV). 2-W vehicles make up the largest market stock (~78%)[11]. Over the past decades, the share of the two-stroke engines (high CO emitters) in 2-W and 3-W vehicles has drastically reduced, from 80% in 1990s to <5% post-2010[12]. Thus, the overall traffic-CO signature is most likely dependent on other factors, for instance, vehicle age.

A category of vehicles across all vintages that contribute disproportionately to pollutant emissions, known as superemitters, is established as 20% of all vehicle fleet (as a function of vehicle age) for South Asia[13]. Given the size of the fleet in this region, the superemitters account for as much as 20 million in 2-W and 4-W each, ~1.5 million in LDDVs and HDDVs[10]. This is much higher than in some parts of the US and Europe. For 2-W a large fraction of the fleet (~50%) is found to be older than 10 years in terms of vehicle age. This is slightly lower for 4-W and HDDVs. The overall average fleet age of vehicles in South Asia (~13 years) is much higher than in countries in Europe and N. America[10]. A higher $CO:CO_2$ ratio is found with increasing fleet age[14], implying that the superemitters might have a much larger role in defining the overall traffic CO isotopic fingerprint in South Asia.

The largest share of $PM_{2.5}$ (upto 75%) and black carbon (BC; upto 95%) emissions is attributed to diesel vehicles in South Asia[10]. A major chunk of this share is indeed found to be from HDDVs and superemitters (up to 80%). Using a conservative BC/CO ratio (0.01 $\mu g\ m^{-3}/\ \mu g\ m^{-3}$), we find the total CO emissions from HDDVs and superemitters alone to be as high as ~14 Tg/yr, an overwhelming portion of the total fossil fuel combustion-derived CO estimate (~15.5 Tg/yr)[15]. This implies that diesel vehicles likely dominate the South Asian CO fingerprint from traffic emissions and possibly from the overall combustion of fossil fuel, respectively. As discussed in Note S1, the diesel engines are found to have distinct isotopic signatures compared to petrol engines, related to a more complete combustion process. Given that there are no isotopic studies of vehicular CO emissions from South Asia, we here establish the fossil fuel combustion endmember by averaging the mean of all fleet-based vehicular emission studies worldwide: $\delta^{13}C$= -27.8±1.5‰; $\delta^{18}O$=19.2±4.9‰ (see Table S4). In this approach, we find the derived $\delta^{18}O$ is indeed closer to the $C^{18}O$ found in diesel emissions, and thus representative of South Asian traffic emissions[1,3]. We do not take the individually tested stationary vehicles (including testbench emissions) into account due to the wide range of isotopic signatures, inconsistent behaviour among vehicles and a lack of established systematic drivers for the observed emission signatures reported in literature.

## Note S3. A discussion on the isotopic fractionation of CO produced in biomass burning

Only a handful of studies so far have investigated the isotopic composition of CO in biomass burning emissions[7,17-19]. Results from controlled burn experiments and ambient samples have differed in magnitude of isotopic fractionation. Typically, fractionation effects are different for two main type of plants with distinct isotopic compositions i.e., $C_3$ and $C_4$ plants. $\delta^{13}C$ is on average -27.1±0.2‰ for $C_3$ plants and -13±1.2‰ for $C_4$ plants[16]. The changes in the isotopic signatures with evolution of emissions in fire depend on several parameters such as combustion efficiency, burning phases, fuel moisture content.

The modified combustion efficiency (MCE) is defined as the portion of $CO_2$, the end-product of combustion, in the total carbon emissions: MCE= $\Delta CO_2/(\Delta CO_2+\Delta CO)$. A strong positive correlation exists between CO isotopic ratios and MCE. The more efficient the combustion (higher MCE), the heavier the emitted CO in terms of both carbon and oxygen isotopic composition. With MCE of 99.8%, CO in biomass samples reach a $\delta^{13}C$ of ~-11‰ and $\delta^{18}O$ of ~+35‰. However, the changes in $\delta^{18}O$ are much larger than in $\delta^{13}C$ with increasing MCE. The isotope ratios are relatively stable below MCE of 80-90%, however, show a sharp increase beyond this mark[17].

A strong decreasing trend has been observed in both isotopes over time from ignition. With a 30% drop in MCE the difference/depletion in $\delta^{13}C$ and $\delta^{18}O$ can be up to ~ -18‰ and ~21‰, respectively. Two clear groups of isotopic clusters are evident for CO based on burning phases: flaming and smoldering[17,18]. The flaming phase (defined as ~96±4% MCE) is accompanied by an enrichment of ~6‰ in $^{13}C$ relative to the $\delta^{13}C$ of the fuel (~25.5‰) . The isotopic fractionation is weaker in the smoldering phase (defined as ~87±6% MCE) with a depletion of ~2‰ in $^{13}C$ relative to the fuel. A similar trend is found in $\delta^{18}O$. However, compared to atmospheric $O_2$ ($\delta^{18}O$ =23.9‰), $^{18}O$ is found to be ~5‰ enriched in the flaming phase and ~9‰ depleted in the smoldering phase[17]. While the $^{13}C$ is close to that of the plant material in initial starting phase, the $^{18}O$ partly depends on oxygen isotopic composition in plant cellulose which is determined by that of meteoric water and highly dependent on relative humidity[18]. The proportion of oxygen in CO from atmosphere and fuel content still remains unclear. Hence the $\delta^{18}O$ in biomass burning may show large variations regionally.

Weak correlations between fuel moisture content and isotopic ratios have been found in indoor burn experiments, implying that dry and wet fuel-type would not show any difference in MCE. When taken together, the average isotopic composition during a whole combustion process is found to be closer to the smoldering phase emitted CO (i.e., depleted in $\delta^{13}C$ and $\delta^{18}O$). This is also supported to ambient observations e.g., during wildfires in USA[7] and biomass burning influenced winter season in Europe[19].

Both $C_3$ and $C_4$ plants have shown similar characteristics during combustion. The derived $\delta^{13}C$ in CO from $C_4$ plant burning is clearly more isotopically enriched than from $C_3$ plant burning, however, the $\delta^{18}O$ in CO are found to be overlapping (see Table S4). The $C^{18}O$ emitted in flaming phase of $C_4$ plant burning are found to be similar to $C^{18}O$ from smoldering phase of $C_3$ plant burning. Given this complexity it is not always possible to distinguish $C_3$ and $C_4$ burning by CO isotopes.

In South Asia, the biomass burnt can be classified into two sectors: open burning (crop residue, forest fires, garbage), domestic burning (agricultural residues, firewood, dung cake)[20]. While $C_3$ and $C_4$ plant burning would contribute with certain proportions, it is challenging to estimate the isotopic signatures of CO emitted from each of these activities in their respective sectors. A lack of CO isotopic studies in this region further complicates the matter.

Hence, we established the South Asian $C_3$-biomass burning isotopic endmember by taking the average of isotopic signatures obtained in all biomass burning related CO studies $\delta^{13}C$: -26.9±4.9‰; $\delta^{18}O$=16.3±5.1‰ (see Table S4). This way we are able to account for most biomass types. This endmember is within the range of CO isotopes in the smoldering phase (characterized in indoor burn experiments) and thus representative for overall combustion process. Similarly, the $C_4$-biomass burning endmember was established to be $\delta^{13}C$: -14.0±3.8‰; $\delta^{18}O$=20.2±4.9‰ (see Table S4).

## Note S4. A discussion on the Keeling-plot approach

A special case of the isotope mass balance is a gas sample taken as the mixture of two sources, namely background air ($\delta_{bgd}$, $m_{bgd}$), and a pollutant ($\delta_p$, $m_p$). The isotope ratio of the gas mixture is then given by

$$\delta_{\mathrm{mixture}} \approx \frac{\mathrm{m_{bgd}}\delta_{\mathrm{bgd}} + \mathrm{m_p}\delta_{\mathrm{p}}}{\mathrm{m_{bgd}} + \mathrm{m_p}} \tag{1}$$

This equation can be rearranged as:

$\delta_{mixture}$ =

$$\frac{\mathrm{m_{bgd}}\ \delta_{\mathrm{bgd}} + (\mathrm{m_{mixture}} - \mathrm{m_{bgd}})\ \delta_{\mathrm{p}}}{\mathrm{m_{mixture}}} = \frac{\mathrm{m_{bgd}}\ (\delta_{\mathrm{bgd}} - \delta_{\mathrm{p}})}{\mathrm{m_{mixture}}} + \delta_{\mathrm{p}} \tag{2}$$

Taking the background concentration, the background isotope ratio, and the isotopic composition of the pollutant as constant, Eq. (2) is linear in $1/m_{mixture}$ with y-intercept $\delta_p$:

$$\delta_{\mathrm{mixture}} \approx \frac{\mathrm{const.}}{m_{\mathrm{mixture}}} + \delta_p \tag{3}$$

This equation is very useful because it enables deriving the isotope value of the “pure” pollutant ($\delta_p$) from a regression of the measured isotope ratios of air samples as a function of the inverse of the measured concentrations, without any further knowledge required about mixing ratios or isotope ratio of the background[21]. With some limitations, Eq. (3) is also applicable for more complex mixtures, assuming e.g. the pollutant to be itself a mixture of two pollutants. The y-intercept can then be interpreted as the isotope ratio of the pollutant mixture, but the linearity of Eq. (3) does not strictly hold when the pollutant composition is variable.

## Note S5. A discussion on the background CO levels at MCOH

By definition, "background" CO signal at MCOH would refer to the [CO] encountered in periods devoid of continental influence and/or long-range transport. Given that MCOH receives air masses spanning a large geographical domain, the [$CO_{background}$] would vary both temporally and seasonally. Apart from this, the isotopic composition of the $CO_{background}$ would also vary with the kinetic isotope effect (KIE) induced due to atmospheric oxidation by hydroxyl radicals (OH·). It is thus important to establish the [$CO_{background}$] first for the current period of interest and then discuss the implications of scavenging process on the signal (details in Note S6).

The histogram of [CO] at MCOH for the winter campaign (see Figure S3 b) shows that the lowest [CO] is estimated to be ~70 to 75 ppb (as established by 5% percentile). This also corresponds to periods with the lowest black carbon (BC) concentrations as well lowest particle count suggesting limited influence from the continental outflow (see Figure S3 a). Coupled to air mass analysis (see Figure S4) it further suggests that such low values were observed during the marine air mass regime and thus are representative of the background levels for this period. In addition, this is corroborated with observations of surface CO in the adjoining oceanic region in the N. Indian Ocean where similar [CO] (~60 to ~90 ppb) have been reported on several different occasions in marine air masses during winter[22]. Furthermore, other remote sites with long term CO measurements have recorded similar [$CO_{background}$][19,23].

The Keeling plot for the CO isotopic signatures at MCOH shows a strong correlation ($R^2 > 0.8$; $p<0.01$) for both stable isotopes ($\delta^{13}C$ and $\delta^{18}O$) (see Fig. 2 in main manuscript), and hence the 2-box mixing model is viable for the South Asian context. The background isotopic signatures from this mixing line are ~ -30.5±0.5‰ for $\delta^{13}C$ and ~-0.8±0.7‰ for $\delta^{18}O$ ([CO]~ 70 to 75 ppb). However, a higher background signal would imply a larger contribution to the overall mixing ratios at MCOH or when apportioned for the contribution to the S $Asia_{source}$ (see Fig. 3 in main manuscript). Hence, we exercise caution in choosing the background, and based on observed changes in the ancillary aerosol parameters (in line with previous observations in the region) find it reasonable to assume the value of ~70 to 75 ppb.

**Note S6. A theoretical model accounting for the effect of scavenging process on the background CO signal at MCOH**

CO is scavenged from the atmosphere mainly by homogeneous gas phase oxidation reaction with OH· radicals. This reaction also induces a kinetic isotopic fractionation (KIE) which plays an important role in altering the isotopic signature of CO emitted from various sources. Here we develop a relationship between the isotopic composition of background CO ($CO_{back,MCOH}$; *intercepted at MCOH*) and the background source signature ($CO_{back,source}$ ; *the initial starting signal of the background*) by accounting for the fractionation effect (KIE) of the scavenging processes, in particular the reaction with OH·. Assuming steady-state, we have:

$$J = k \cdot [CO] \tag{4}$$

J = CO flux (e.g., g m$^{-3}$ s$^{-1}$); k = reaction rate coefficient (e.g., s$^{-1}$); [CO] = CO concentration (e.g., g m$^{-3}$).

The isotope-ratio (R) can be expressed as:

$$R = \frac{[CO_{heavy}]}{[CO_{light}]} = \frac{J_{heavy}/k_{heavy}}{J_{light}/k_{light}} \tag{5}$$

The kinetic isotope effect (KIE) is defined as:

$$KIE \equiv \frac{k_{light}}{k_{heavy}} \tag{6}$$

The isotope-ratio of *the source* can be estimated as the ratio of the fluxes:

$$R_{source} = \frac{F_{heavy}}{F_{light}} \tag{7}$$

The observed isotope-ratio then relates to the source ratio and KIE as:

$$R_{obs} = R_{source} \cdot KIE \tag{8}$$

Introducing the δ-scale (in per mill, where std is the isotope-ratio of the *standard*):

$$(\delta_{source} + 1) \cdot R_{std} = (\delta_{obs} + 1) \cdot R_{std} \cdot \left(\frac{1}{KIE}\right) \tag{9}$$

Setting $\delta_{source}$ is the CO isotopic signature of the background source (in this case $CO_{back,source}$) and $\delta_{obs}$ is the measured CO isotopic signature in ambient air at the sampling site (in this case $CO_{back,MCOH}$)

and rearranging:

$$\delta_{back,source} + 1 = (\delta_{back,MCOH} + 1) \cdot \left(\frac{1}{KIE}\right) = \delta_{back,MCOH} + (\delta_{back,MCOH} + 1) \cdot \left(\frac{1}{KIE} - 1\right) + 1 \tag{10}$$

$$\delta_{back,source} - \delta_{back,MCOH} = (\delta_{back,MCOH} + 1) \cdot \left(\frac{1}{KIE} - 1\right) \tag{11}$$

The effect of isotopic substitution on the competition between the forward and backward reactions of the vibrationally excited reaction intermediate of CO+OH· → ($HOCO^{\#}$) is subtle, since the reaction leads to a positive KIE (enrichment) in $^{13}C^{16}O$ isotope and a negative KIE (depletion) in $^{12}C^{18}O$ isotope[24]. In fact, enrichment of upto ~ +5‰ for $^{13}C^{16}O$ and depletion upto ~ -10‰ for $^{12}C^{18}O$ has been reported for the isotopologues upon a complete reaction, respectively[6]. Based on this and eqn.11, the trajectory of the background signal is estimated and shown in Figure S8. It should be noted that the background signal would move along this line based on the extent of KIE.

## Note S7. Source apportionment based on a hierarchical Bayesian statistical model

The hierarchical Bayesian model accounts for Keeling fit, isotope mass balance and primary endmember distribution. Here, each is discussed in detail and the relevant steps in the formulation of the model are outlined:

*Keeling fit*

The good correlations of both isotope signatures ($\delta^{18}O$: $R^2 = 0.95$ and $\delta^{13}C$: $R^2=0.81$; Figure 2 in the main manuscript) with the inverse CO concentrations (i.e., the Keeling relation), suggests that the isotope variability at MCOH may be described by a two-state mixture: a constant background and a temporally variable source. While the background is likely to be affected by kinetic isotope effects (KIE), this is not expected to be the case for the isotope signature in the limit of $[CO]\rightarrow\infty$, as this would correspond to the “source” signature. As such, the values in this limit ($\delta^{18}O_{SAsia}$; $\delta^{13}C_{SAsia}$ ; Figure 3 in the main manuscript) reflects the isotopic source signature of the continental CO emissions. The Keeling relations may be expressed as (index i signifies data point):

$$\delta^{18}O(i) = \frac{k_{18}}{[CO(i)]} + \delta^{18}O_{SAsia} \quad (12a)$$

$$\delta^{13}C(i) = \frac{k_{13}}{[CO(i)]} + \delta^{13}C_{SAsia} \quad (12b)$$

Where the slopes, ($k_{18O}$; $k_{13C}$), are fitted along with the $\delta^{18}O_{SAsia}$; $\delta^{13}C_{SAsia}$. Within a Bayesian linear regression framework, prior information regarding the slopes and offset may be represented by uniform distributions, while prior for the estimator of variability around the mean fit was set to inverse gamma, and the posterior was assumed normal. The South Asian isotopic signature is established directly through this fit, while the background signature may be calculated using an estimate of the background CO concentrations (~70ppb for MCOH; Note S5).

*Isotopic mass balance*

In this study, our aim is to estimate the fractional (*f*) contributions from primary and secondary CO to the South Asian continental CO emission. Assuming isotopic mass balance, we have:

$$\delta^{18}O_{SAsia} = f_{primary}\delta^{18}O_{primary} + f_{secondary}\delta^{18}O_{secondary} \quad (13a)$$

$$\delta^{13}C_{SAsia} = f_{primary}\delta^{13}C_{primary} + f_{secondary}\delta^{13}C_{secondary} \quad (13b)$$

Where observed isotope signature is the weighted sum of the isotopic signatures of the two sources; the endmembers ($\delta^{13}C_{primary}$ and $\delta^{13}C_{secondary}$; $\delta^{18}O_{primary}$ and $\delta^{18}O_{secondary}$;).

Combined with Eqn. (12), we obtain a method for constraining the relative contributions of primary and secondary CO by combining all individual isotope and concentration data points:

$$\delta^{18}O(i) - \frac{k_{18}}{[CO(i)]} = f_{primary}\delta^{18}O_{primary} + f_{secondary}\delta^{18}O_{secondary} \quad (14a)$$

$$\delta^{13}C(i) - \frac{k_{13}}{[CO(i)]} = f_{primary}\delta^{13}C_{primary} + f_{secondary}\delta^{13}C_{secondary} \quad (14b)$$

As discussed in the main manuscript, the secondary CO endmember is same as that of the NMHC-oxidation source, while the primary CO endmember is a combination of several sources. For S Asia these mainly include incomplete combustion from $C_3$ plants, $C_4$ plants and fossil fuels. The primary endmember values may be written as:

$$\delta^{18}O_{primary} = f_{C3}\delta^{18}O_{C3} + f_{C4}\delta^{18}O_{C4} + f_{fossil}\delta^{18}O_{fossil} \quad (15a)$$

$$\delta^{13}C_{primary} = f_{C3}\delta^{13}C_{C3} + f_{C4}\delta^{13}C_{C4} + f_{fossil}\delta^{13}C_{fossil} \quad (15b)$$

Generally, any mass balance-based source apportionment calculation needs to consider, not only the means, but also the variability of the endmembers when estimating the fractional source contributions. This may be done, e.g., using a Bayesian statistics approach, where the endmember distributions may be represented by normal distributions, where the means and standard deviations are derived from empirical near (pure) source data[25]. The multiple data points used in Eqn. (14a-b) provides estimation of the variability of the isotope signatures, which constraints the influence of the endmember variability relative observed data, and thereby narrows the variability in the estimates of $f_{\mathrm{primary}}$ and $f_{\mathrm{secondary}}$.

*Primary endmember distribution*

The primary endmember distribution is here represented as a mix of three different primary source components. However, the relative contributions of these are uncertain. But we do have prior information, e.g., from bottom-up emission inventories of primary CO emissions. This information may then be used as a prior in a Bayesian framework. Bayesian calculations of the posterior of fractional, a Dirichlet prior is often employed:

$$p(f_1, f_2, \dots, f_n) = \frac{1}{N(\alpha)} \prod_{i=1}^{n} f_i^{\alpha_i - 1} \quad (16a)$$

Where, f are the fractional source contributions (here n = 3, representing $C_3$, $C_4$ and fossil) and,

N(α) is the normalizing function:

$$N(\alpha) = \frac{\prod_{i=1}^{n} \Gamma(\alpha_i)}{\Gamma(\sum_{i=1}^{n} \Gamma(\alpha_i))} \quad (16b)$$

Where Γ is the gamma function.

The degree of prior information is encoded in the exponents, $\alpha_i$. If all alphas are set to one, we have a flat prior, essentially no prior information. The mean relative contribution for a component i ($\mu_i$), is related to the exponents as:

$$\mu_i = \frac{\alpha_i}{\sum_{k=1}^{n} \alpha_k} \quad (17)$$

Thus, one may directly connect the exponents to the prior knowledge, e.g., from the bottom-up emission inventory.

However, Eq. (17) is an under-determined system in itself: we cannot un-ambiguously solve for $\alpha_I$ using only known means. To estimate the alphas, we therefore need additional constraints.

The variance ($\sigma_\iota^2$) for the dimensions i of a Dirichlet distribution is given by:

$$\sigma_i^2 = \frac{\mu_i(1-\mu_i)}{\sum_{k=1}^{n} \alpha_k + 1} \quad (18)$$

We note that the variance for each individual dimension, i, as well as the total variance ($\sum_{k=1}^{n} \sigma_k^2$), depend on $1/(\sum_{k=1}^{n} \alpha_k + 1)$. Since bottom-up emission inventories often report high uncertainties (e.g. 125% or higher)[47], and since such uncertainty estimates are also often not available/estimated, we here use a prior with minimum prior constraints. The least informed prior, while still retaining prior information regarding mean contributions, should then maximize the variance; minimize the sum of $\alpha_i$. We here assume that the prior distribution is mono-modal. This means $\alpha_i \geq 1$, for all i, while $\alpha_k > 1$ for at least one k. We then have the following optimization problem: we need to find the smallest sum of alphas, such that all values are equal or larger than one. The smallest sum is obtained when at least one of the alphas equals 1. Since all other alphas need to be equal or larger than 1, this means that the alpha that equals one ($\alpha_k = 1$) need to correspond to the smallest mean ($\mu_k = \min(\mu_1, \mu_2, \ldots, \mu_n,)$). We arrive at the following parametrization:

$$\alpha_i = \frac{\mu_i}{\min(\mu_1, \mu_2, \ldots, \mu_n)} \quad (19)$$

We note that in this formulation with three dimensions, $\mu_i = 1/3$ for all i, equates to $\alpha_I = 1$ for all i; the un-informed prior (unit simplex).

_Posterior probability density function_

The Bayesian model used here to compute the relative contributions from primary and secondary sources to CO in the South Asian continental emissions then relies on a number of modules, that are combined into the model. We can summarize the posterior probability density function as:

$$P(f_{primary} | \delta^{18}O; \delta^{13}C) \sim \prod_{i=1}^{n} \{P(\delta^{18}O_i | f_{primary}; k_{18}; k_{13}; f_{C3}; f_{C4}; f_{fossil}) \cdot P(\delta^{18}O_i | f_{primary}; k_{18}; k_{13}; f_{C3}; f_{C4}; f_{fossil})\} \cdot P(k_{18}; k_{13}) P(f_{C3}; f_{C4}; f_{fossil}) P(f_{primary}) \quad (20)$$

Where, the likelihood:

$$\prod_{i=1}^{n} \{P(\delta^{18}O_i | f_{primary}; k_{18}; k_{13}; f_{C3}; f_{C4}; f_{fossil}) \cdot P(\delta^{18}O_i | f_{primary}; k_{18}; k_{13}; f_{C3}; f_{C4}; f_{fossil})\} \quad (21)$$

Is assumed normal, fulfilling isotopic mass-balance. The constraints provided by the multiple data points (i) are implemented through the product, as in Eqn. (14a-b),

The prior for the slopes: $P(k_{18}; k_{13})$

Was assumed normal, while the estimator for the variability inverse gamma (see above for details).

The prior for the fractional source contributions to the primary endmember: $P(f_{C3}; f_{C4}; f_{fossil})$
Was assumed Dirichlet distributed (see above for details).

And the prior for the fractional contribution of primary: $P(f_{primary})$
Was assumed uniform.

The MCMC simulations were implemented using a Metropolis-Hastings algorithm[50,51], implemented in MATLAB version 2015b code with 1000.000 iterations with an initial burn-in phase of 10.000 (to assure proper annealing prior to estimation of the probability density functions) and a data thinning of 100 (to remove correlations between iterations)[25].

## Note S8. Establishing an informed prior using emission estimates from bottom-up inventories

The main primary sources include burning of $C_3$-biomass (e.g., wood; and agricultural waste burning of wheat and rice), burning of $C_4$-biomass (e.g., agricultural waste burning of sugarcane, millet and maize) and fossil fuel combustion.

Biomass burning can be divided into two categories: crop residue burning and residential/domestic use. For crop residue burning, the fraction of $C_3$- vs $C_4$-plant residue generated, and residue burnt was first estimated and the CO produced from each was apportioned from estimates of total CO[48]. This was ~7 Tg/yr for $C_3$ plants and ~2 Tg/yr for $C_4$ plants.

The total CO estimated from an Asia-specific bottom-up emission inventory[47] ~62 Tg/yr. This was then divided into CO from various sectors and grouped into two categories: Biomass burning-CO and Fossil fuel combustion -CO. The estimates for total biomass burning CO was ~43 Tg/yr and fossil fuel combustion was ~19 Tg/yr.

The crop residue burning generated CO amounted for 16% from $C_3$ plants and 5% from $C_4$ plants. The residential use generated CO amount for 49% and this was allocated to $C_3$ plants as well. Fossil fuel combustion generated CO accounted for 30%. It should be noted that the uncertainty in the emission inventory-based estimates of CO produced for the S Asian region range from ±51% for powerplants to ±192% for domestic sectors, with the total uncertainty amounting to ±114%[47]. Furthermore, we used the estimates for the year 2008 and the growth rate of CO between 2010-2015 is estimated to be ~4% with an uncertainty of ±136% (for 2015)[49]. However, the corresponding changes in the distribution of CO between various sectors is unclear and therefore in this study we relied on the 2008 estimates.

## Supplementary Figures

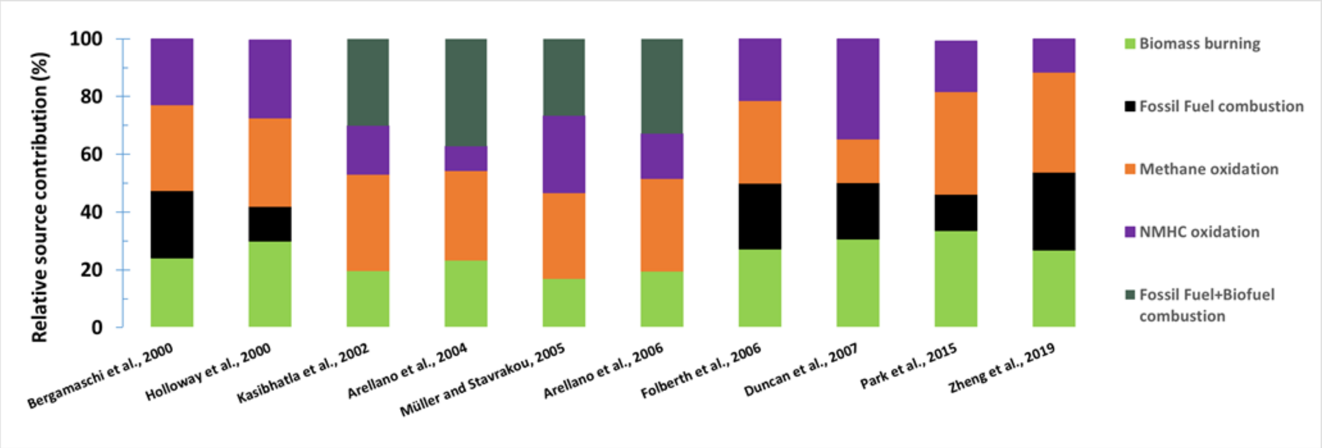


**Figure S1. Fractional contribution of major source categories to global carbon monoxide (CO) budget.** Note that the biomass burning category here includes contribution from open biomass burning ($BB_o$) and biofuel combustion (BF) for all studies other than Kasibhatla et al. 2002; Arellano et al., 2004; Muller and Stavrakou 2005; Arellano et al.,2006. For these four studies only the $BB_o$ fraction is reported separately, and the BF and Fossil Fuel combustion estimates are reported together.

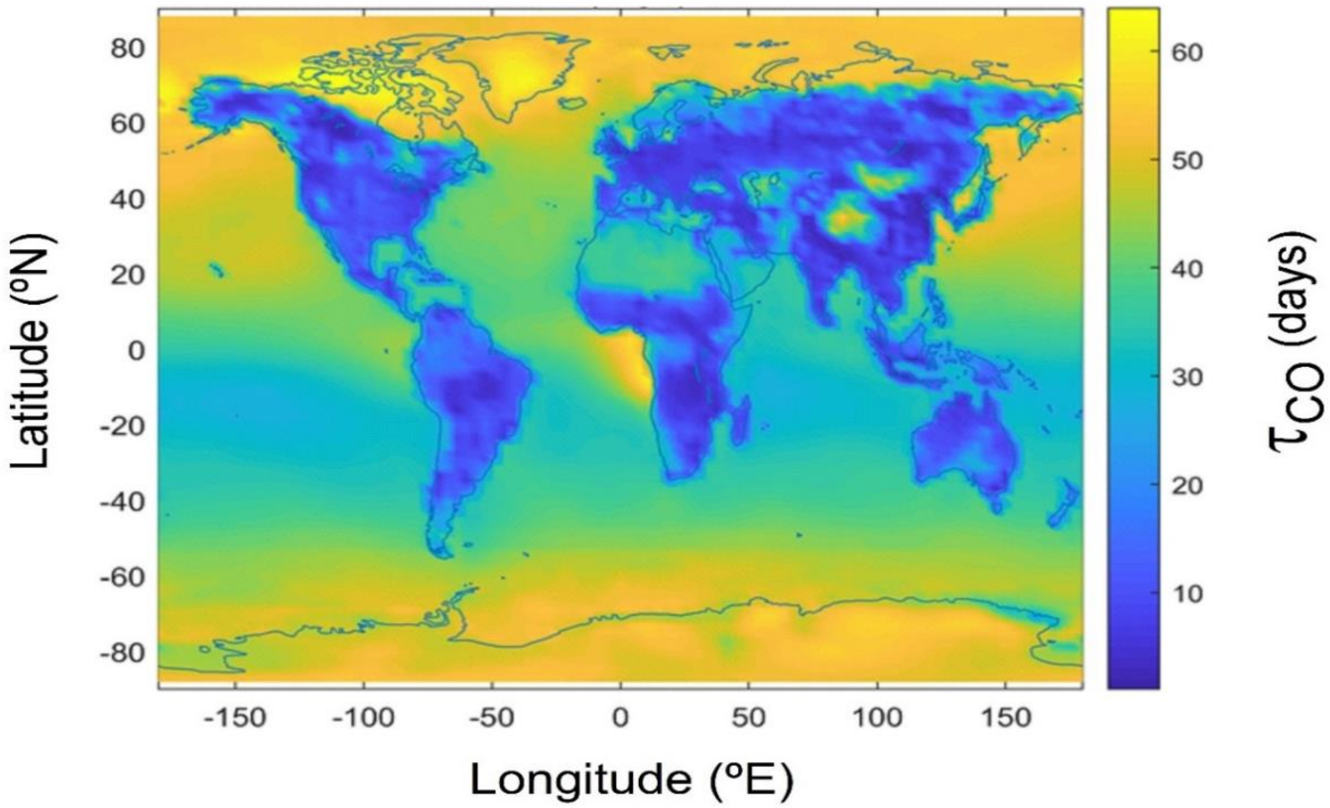


**Figure S2. Wintertime (December to February) lifetime (τ) of carbon monoxide (CO).** This is retrieved from the global inventory of CO[41]. The Zheng et al., inventory uses multi species constraints and a relatively improved resolution and is able to provide trends in CO column as well as a long-term dataset for CO emission flux estimates. It also provides CO chemical production and CO sink estimates. The atmospheric lifetime of CO is derived from this inventory using the ratio of the CO sink estimates and CO emission flux estimates.

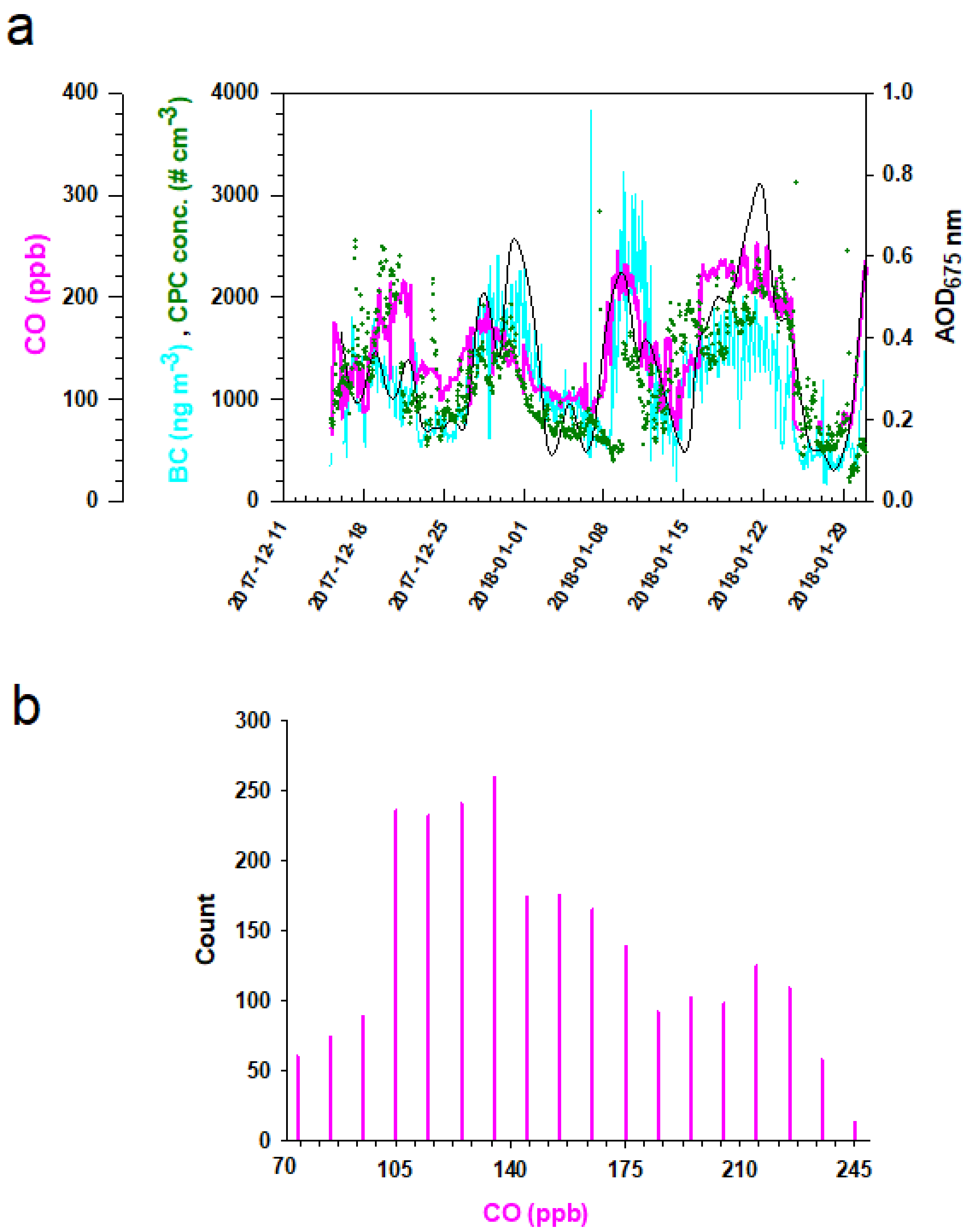


**Figure S3. Online measurements of CO and aerosol parameters at MCOH.** (a) The carbon monoxide (CO), black carbon (BC), particle number concentrations are measured using the HORIBA-APM 375, Aethalometer (AE-33) and Condensation Particle Counter (CPC; TSI Inc. model 3772) respectively. The aerosol optical depth at 675 nm is measured using the CIMEL sun-photometer as part of the NASA AERONET program. (b) Histogram of the online CO concentrations (with 3 min timestep averaged hourly) is shown for the same period as in (a). Note the lowest CO mixing ratio encountered at MCOH is ~70 to 75 ppb.

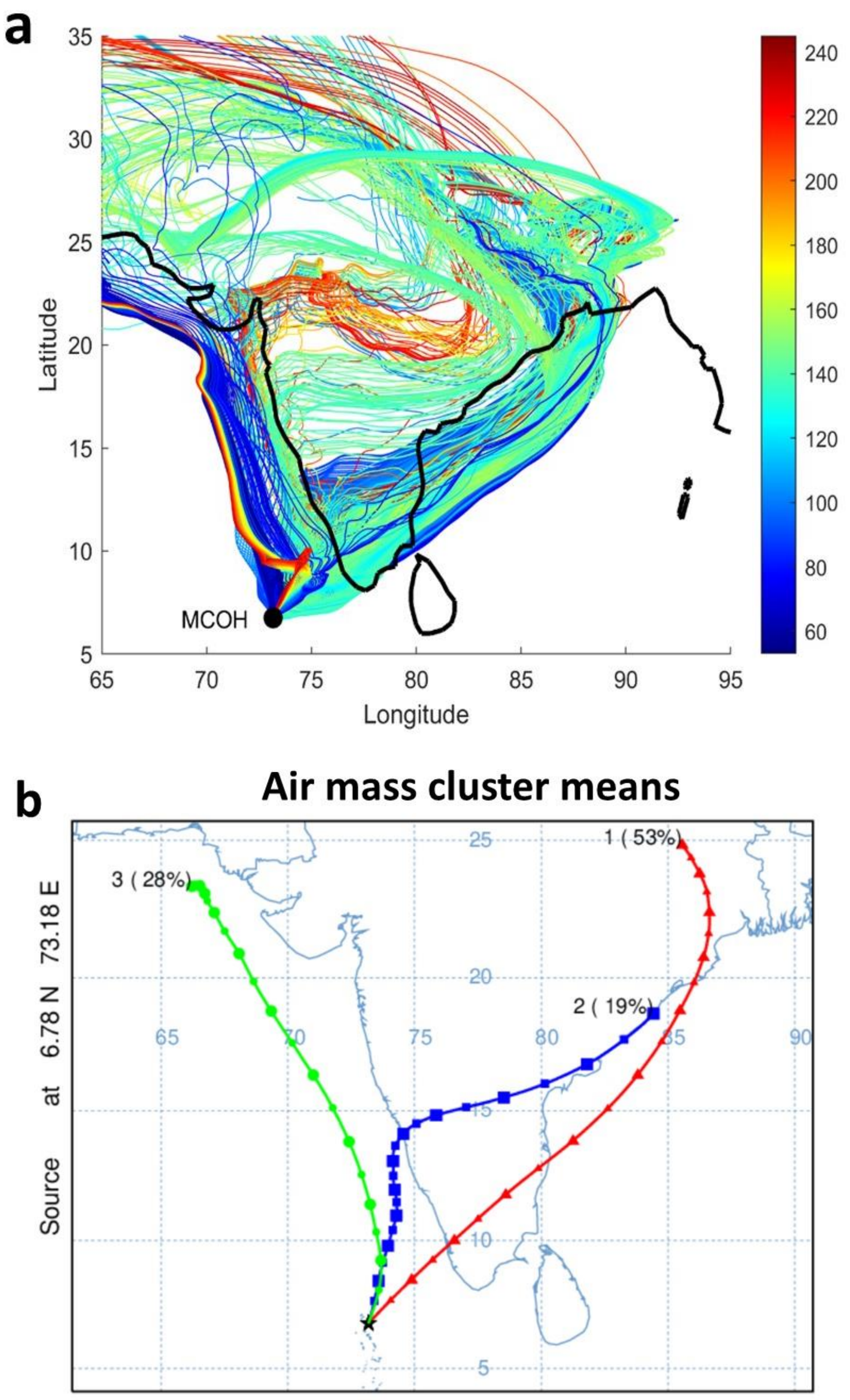


**Figure S4. Air mass transport pathways for CO sampled at MCOH.** (a) 10-day air mass back-trajectories were generated (corresponding to the online CO sampled; see Figure S3a) at Maldives Climate Observatory at Hanimaadhoo (MCOH) with an arrival height of 100 m, computed hourly using NOAA Hybrid Single-Particle

Lagrangian Integrated Trajectory model (HYSPLIT) version 4 for the period 15 December 2017 – 31 January 2018. (b) Air mass cluster analysis conducted for back trajectories in (a) and the cluster means are shown.

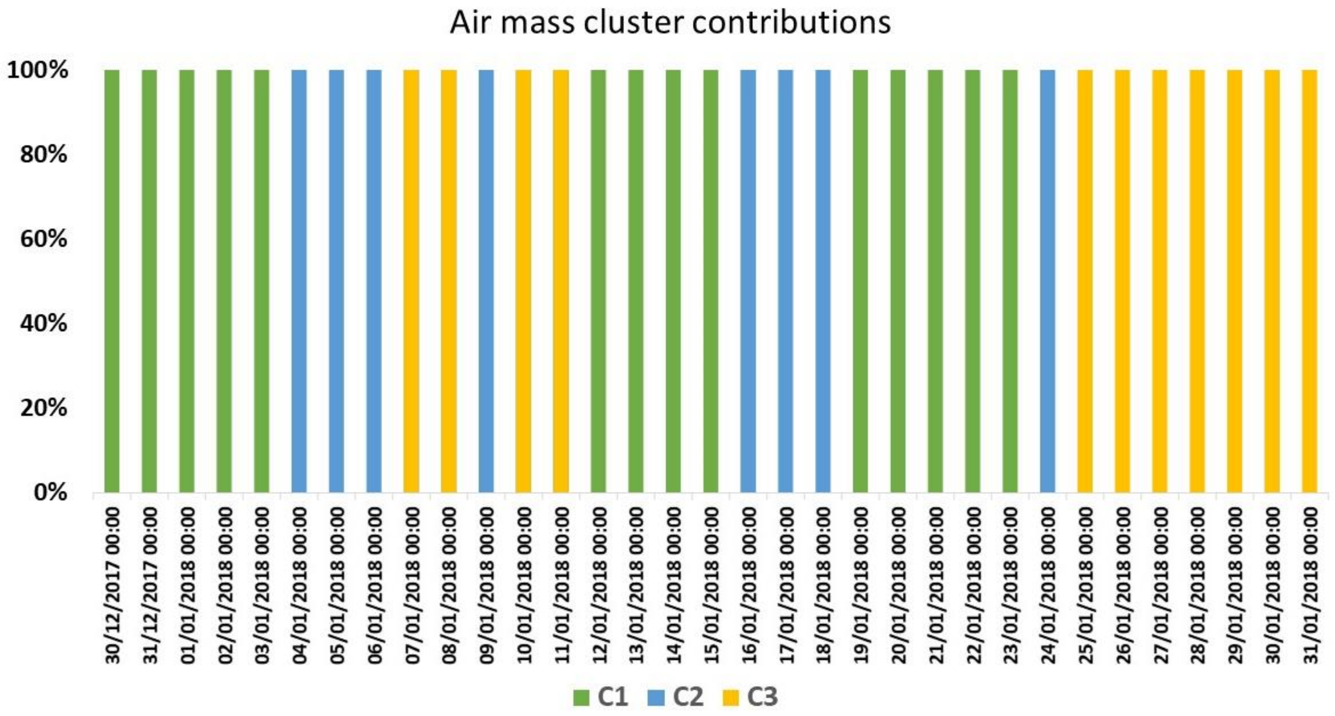


**Figure S5. Air mass fractional cluster contribution.** The fractional cluster contributions are showns for the 3 air mass clusters during the winter campign. Clusters (1, 2, 3) are shown in Figure S4b.

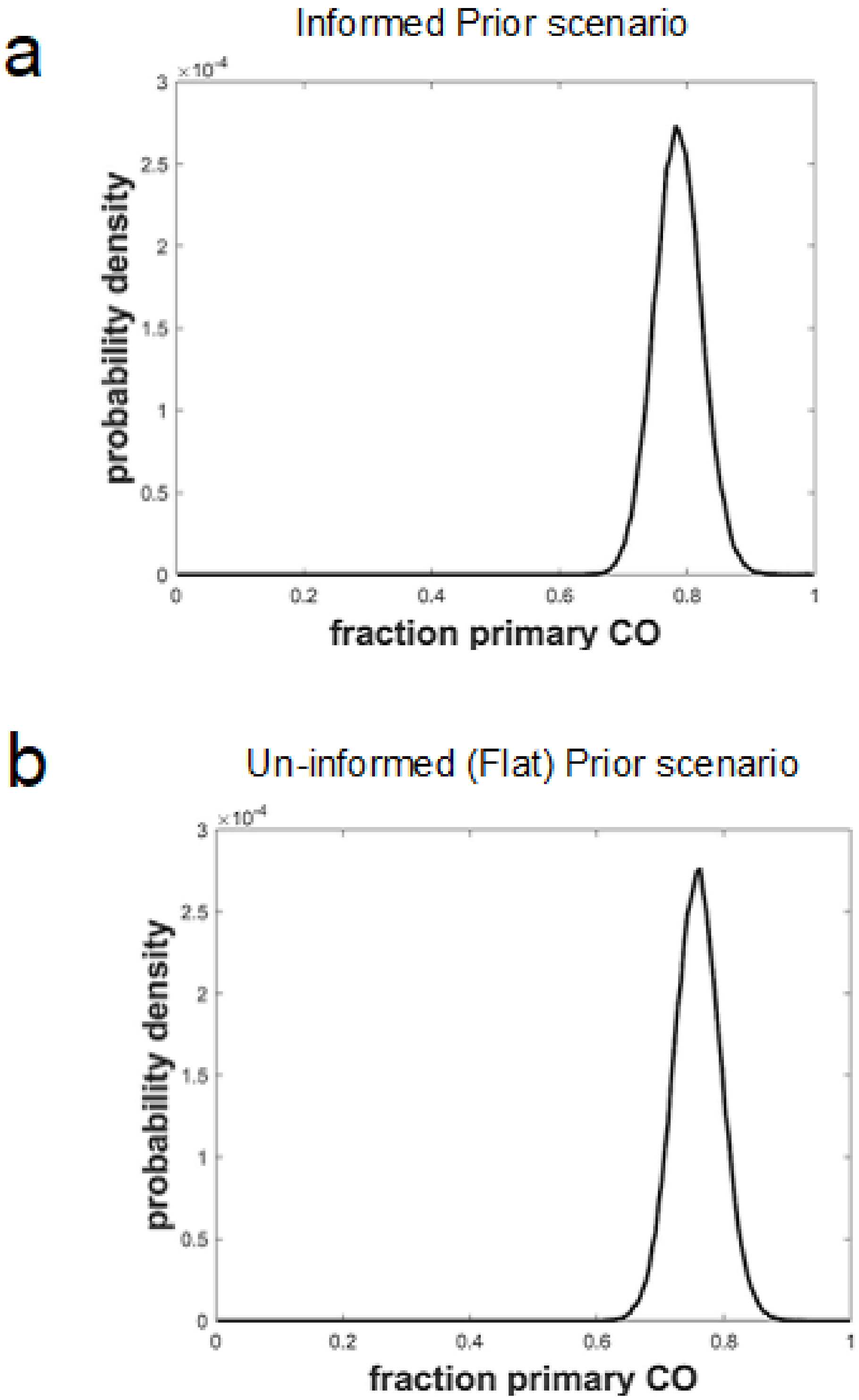


**Figure S6. MCMC-based probability density functions for the fraction primary CO.** The simulations are conducted with a) informed prior, and b) uninformed prior scenarios.

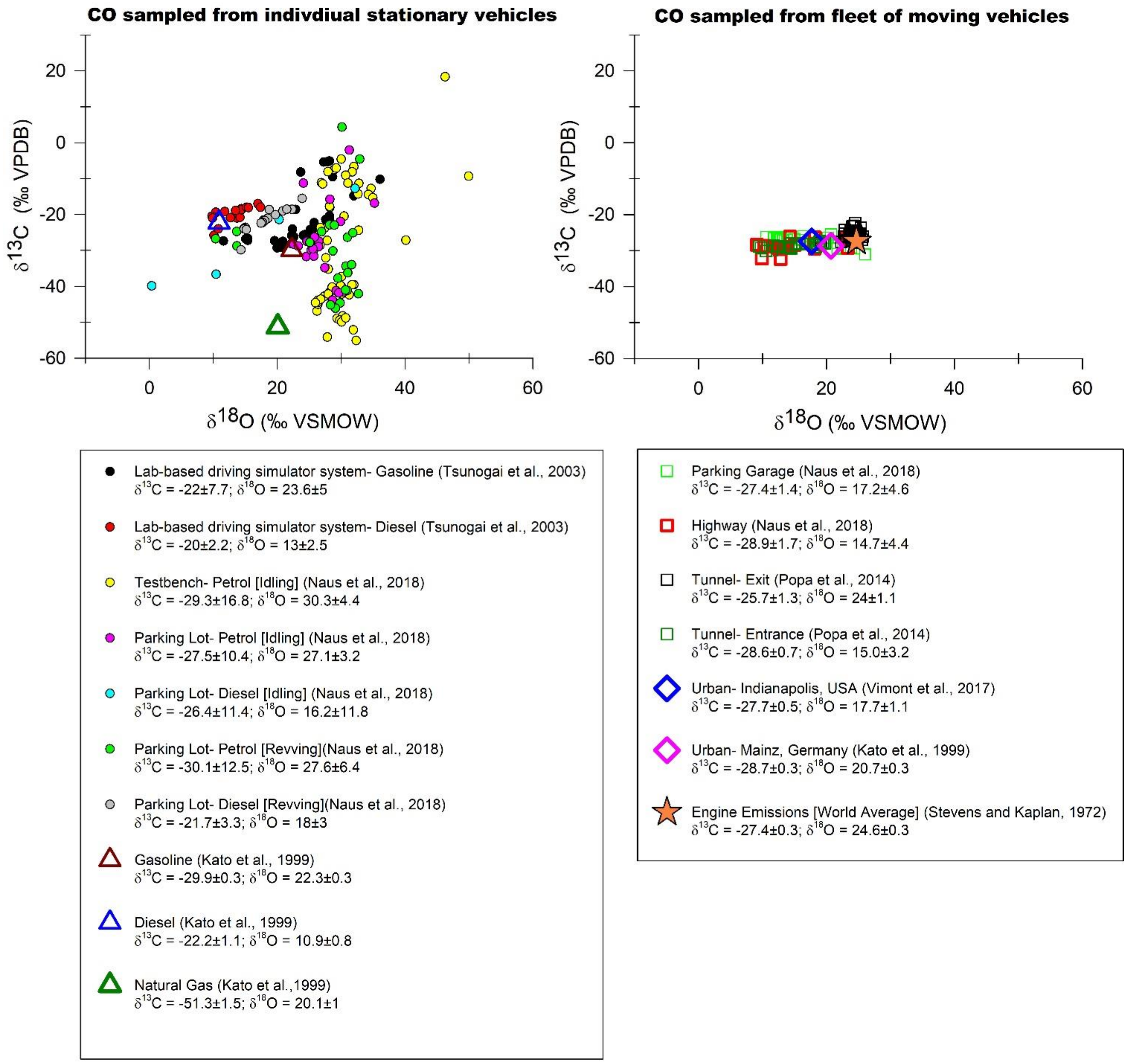


**Figure S7. Stable isotopic signatures of CO from fossil fuel combustion in vehicular emissions.**

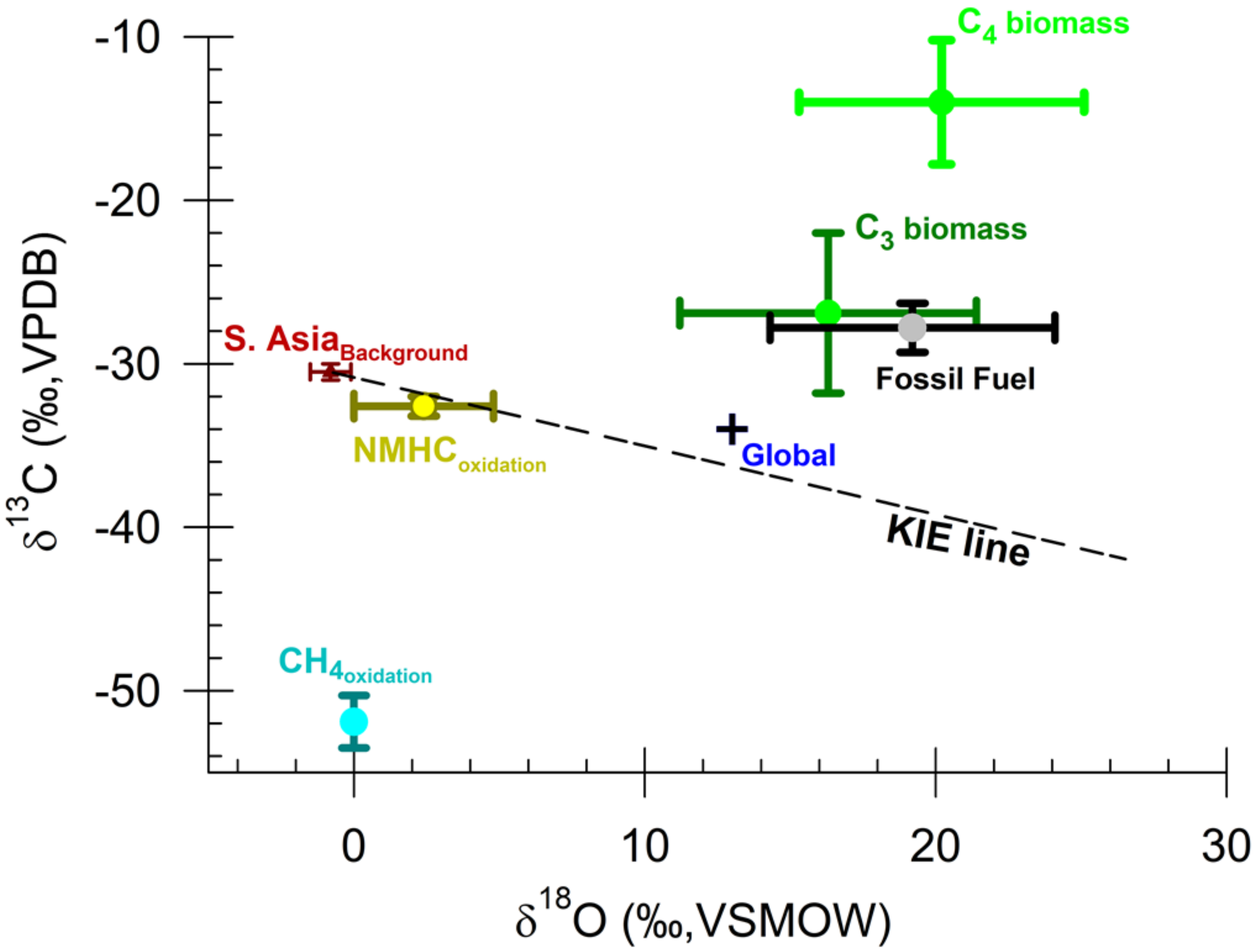


**Figure S8. The trajectory of the background signal at MCOH with reference to the scavenging process (KIE line).** See mathematical formulation in Note S6.

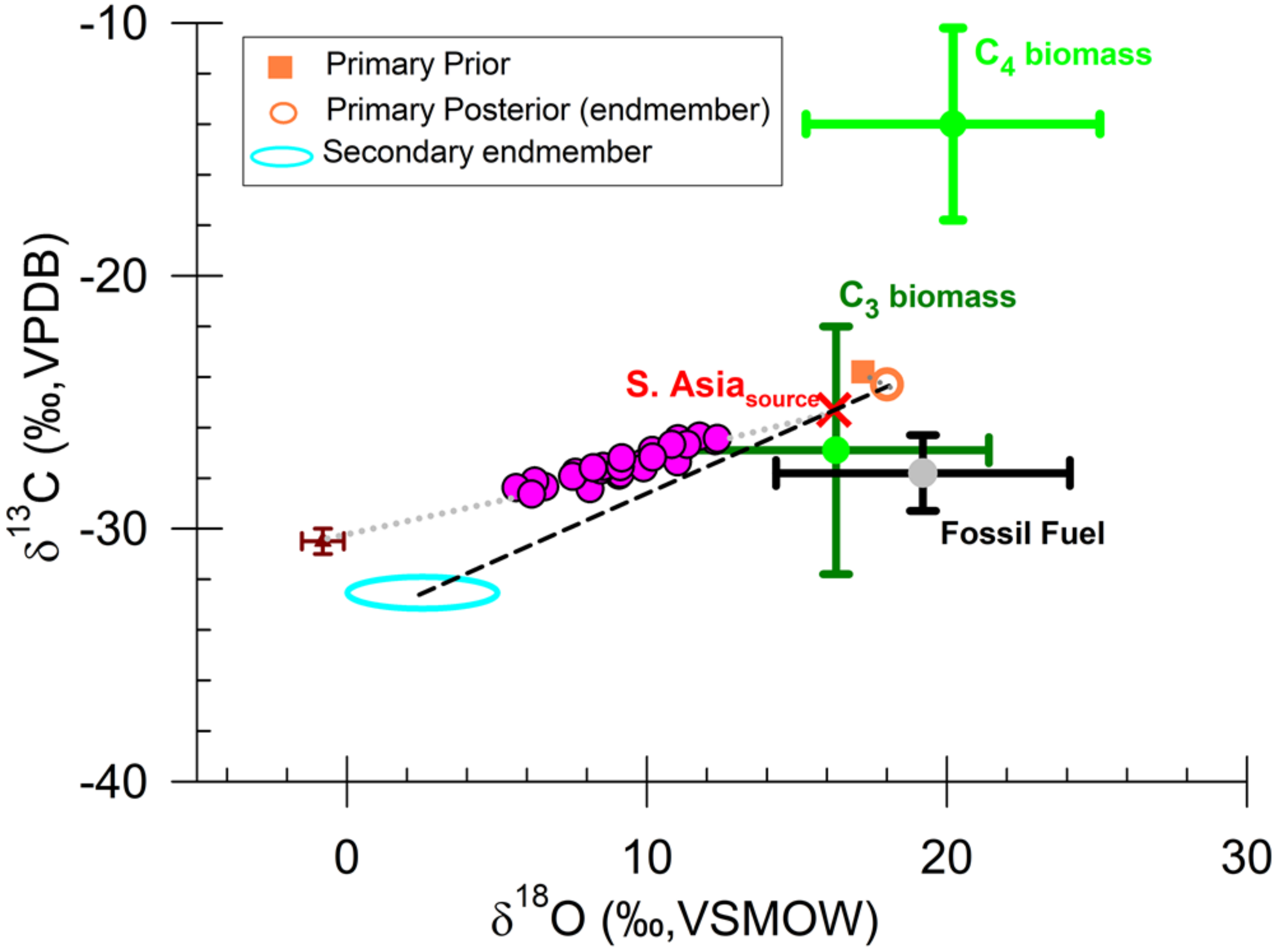


**Figure S9. Bayesian dual-isotope source contribution modeling of CO in South Asia**. The S Asian$_{source}$ signal (see Figure 3 in the main manuscript) is apportioned using endmembers for primary CO and secondary CO. The primary endmember (orange, open circle) is deduced from un-informed prior (where the contribution of the three primary sources are assumed to be equal). The secondary CO endmember (cyan, ellipse) is the same as the NMHC$_{oxidation}$ source (see main manuscript; see also Table S1).

## Supplementary Tables

**Table S1. Dual-isotope [carbon ($\delta^{13}C$) and oxygen ($\delta^{18}O$)] endmembers for different CO sources [*Primary origin*: $C_3$ biomass burning, $C_4$ biomass burning, fossil fuel combustion], [*Secondary origin*: Oxidation of non-methane hydrocarbons (NMHC's) and methane ($CH_4$), respectively].** The choice of endmembers is based on extensive up-to-date database of CO isotopic signatures compiled from the literature (see details in Table S3) and only the Mean ± SD are shown here. Also reported are the isotopic signatures for background CO (see Note S5; Figure S8). A discussion on fractionation effects can be found in Notes S1-S3.

| | $C_3$ biomass | $C_4$ biomass | Fossil fuel | $NMHC_{oxdn.}$ | $CH_{4oxdn.}$ | Background |
|---|---|---|---|---|---|---|
| $\delta^{13}C$ | -26.9±4.9 | -14.0±3.8 | -27.8 ±1.5 | -32.6±0.6 | -51.9±1.6 | -30.5±0.5 |
| $\delta^{18}O$ | +16.3±5.1 | +20.2±4.9 | +19.2±4.9 | +2.4±2.4 | 0 | -0.8±0.7 |

**Table S2. Flask-based sampling of carbon monoxide (CO) at the Maldives Climate Observatory at Hanimaadhoo (MCOH).** The average concentration and average stable isotopic composition ($\delta^{13}C$ and $\delta^{18}O$) of sampled batches (n=2 flasks) are reported corresponding to the sampling date.

| Sampling Date (mm-dd-yyyy) | CO (ppb) | $\delta^{13}C$ (‰) | $\delta^{18}O$ (‰) | 1/CO (ppm) |
|---|---|---|---|---|
| 12/15/2017 | 188 | -27.90 | 9.07 | 5.3 |
| 12/16/2017 | 160 | -28.42 | 8.10 | 6.3 |
| 12/17/2017 | 196 | -27.39 | 9.90 | 5.1 |
| 12/18/2017 | 173 | -27.82 | 9.12 | 5.8 |
| 12/19/2017 | 185 | -27.45 | 9.81 | 5.4 |
| 12/20/2017 | 186 | -27.59 | 9.89 | 5.4 |
| 12/21/2017 | 211 | -26.93 | 10.62 | 4.8 |
| 12/21/2017 | 213 | -27.00 | 11.07 | 4.7 |
| 12/22/2017 | 217 | -27.36 | 11.02 | 4.6 |
| 01/04/2018 | 156 | -27.69 | 8.47 | 6.4 |
| 01/05/2018 | 147 | -27.78 | 7.62 | 6.8 |
| 01/06/2018 | 146 | -27.93 | 7.53 | 6.8 |
| 01/07/2018 | 135 | -28.34 | 6.60 | 7.4 |
| 01/08/2018 | 164 | -27.54 | 8.54 | 6.1 |
| 01/09/2018 | 248 | -26.52 | 12.27 | 4.0 |
| 01/10/2018 | 269 | -26.35 | 11.75 | 3.7 |
| 01/11/2018 | 235 | -26.45 | 11.04 | 4.2 |
| 01/12/2018 | 180 | -26.91 | 10.18 | 5.6 |
| 01/13/2018 | 140 | -27.60 | 8.21 | 7.1 |
| 01/14/2018 | 158 | -27.55 | 9.12 | 6.3 |
| 01/15/2018 | 156 | -27.20 | 9.16 | 6.4 |
| 01/20/2018 | 282 | -26.44 | 12.35 | 3.5 |
| 01/21/2018 | 259 | -26.67 | 11.34 | 3.9 |
| 01/22/2018 | 240 | -26.67 | 10.83 | 4.2 |
| 01/23/2018 | 183 | -27.17 | 10.19 | 5.9 |
| 01/24/2018 | 117 | -28.12 | 6.26 | 8.5 |
| 01/25/2018 | 107 | -28.38 | 5.64 | 9.3 |
| 01/26/2018 | 115 | -28.64 | 6.16 | 8.7 |

**Table S3. A compilation of the isotopic signatures [ ($\delta^{13}C$, $\delta^{18}O$)] of carbon monoxide for different sources [$C_3$ biomass burning, $C_4$ biomass burning, fossil fuel combustion, oxidation of non-methane hydrocarbons (NMHC's) and methane ($CH_4$)].** This table shows the data used for compiling the source-specific endmembers used in the current study (in Table S1), which is a censored version of the full dataset. The censoring is primarily done for fossil fuel combustion- and Biomass burning-CO isotopic signatures and is based on the arguments in Note S1-S3 (see also Fig. S7). The dataset with all "raw" data (complied from all published CO studies) will be available on the Bolin Centre Database (http://bolin.su.se/data/Dasari-2021). Note that the uncertainties in the isotopic signatures as well as the isotopic fractionation reported for the corresponding measurements is accounted in this compiled version.

| $C_3$ biomass burning | | $C_4$ biomass burning | | Fossil fuel combustion | | $NMHC_{oxdn.}$ | | $CH_{4\ oxdn.}$ | |
|---|---|---|---|---|---|---|---|---|---|
| $\delta^{13}C$‰ | $\delta^{18}O$‰ | $\delta^{13}C$‰ | $\delta^{18}O$‰ | $\delta^{13}C$‰ | $\delta^{18}O$‰ | $\delta^{13}C$‰ | $\delta^{18}O$‰ | $\delta^{13}C$‰ | $\delta^{18}O$‰ |
| -28.2[a*] | 28.5[a*] | -17.8 [a§] | 25.6 [a§] | -28.8[f*] | 21.8[f*] | -33.3[k] | 4.8[k] | -50[l] | 0[l] |
| -18.6 [a*] | 13.1[a*] | -14 [a§] | 18.8 [a§] | -26[f*] | 12.6[f*] | -32.3[k] | 2.4[k] | -53[m] | 0[m] |
| -32.6 [a#] | 18[a#] | -10.2[e¤] | 16.2 [e¤] | -30.6[f#] | 19.1[f#] | -32.20[c] | 0[c] | -52.6[n] | 0[n] |
| -27.2 [a#] | 12.2[a#] | | | -27.2[f#] | 10.3[f#] | | | | |
| -24.7[b] | 16.3[b] | | | -28.2[g] | 18.8[g] | | | | |
| -22.9[c] | 17.2[c] | | | -27.2[g] | 16.6[g] | | | | |
| -21.3[d] | 16.3[d] | | | -27[h*] | 25.5[h*] | | | | |
| -29.7[e*] | 18.4 [e*] | | | -24.4[h*] | 22.9[h*] | | | | |
| -29.9 [e#] | 12.0 [e#] | | | -29.3[h#] | 18.2[h#] | | | | |
| -33.8 [e§] | 10.9 [e§] | | | -27.9[h#] | 11.8[h#] | | | | |
| | | | | -29[i] | 21[i] | | | | |
| | | | | -28.5[i] | 20.4[i] | | | | |
| | | | | -27.7[j] | 24.9[j] | | | | |
| | | | | -27.1[j] | 24.3[j] | | | | |

Note: The superscripts in the table refer to these studies: *[a] Qiu, 2019 (Master thesis IMAU, [*]willow wood chips, [#]Amazon forest fires, [§]corn); [b] Saurer et al., 2009 (firewood); [c] Röckmann et al., 2002; [d] Brenninkmeijer et al., 1999; [e] Kato et al., 1999 ([*]Eucalyptus branches, [#]Eucalyptus twigs, [§]Leaves, [¤]Maize); [f] Naus et al., 2018 ([*]Parking garage, [#]Highway); [g] Vimont et al., 2017 (urban traffic :: USA); [h] Popa et al., 2014 ([*]tunnel exit, [#]tunnel entrance :: Switzerland); [i] Kato et al., 1999 (Urban traffic :: Germany); [j] Stevens et al., 1972 (World Engines); [k] Vimont et al., 2019; [l] Bergamaschi et al., 2000 ; [m] Brenninkmeijer and Röckmann 1997 ; [n] Cantrell et al., 1990*

## References


1. Tsunogai, U.; Hachisu, Y.; Komatsu, D. D.; Nakagawa, F.; Gamo, T.; Akiyama, K. An updated estimation of the stable carbon and oxygen isotopic compositions of automobile CO emissions. *Atmos. Environ.* **2003**, *37*, 4901–4910.

2. Popa, M. E.; Vollmer, M. K.; Jordan, A.; Brand, W. A.; Pathirana, S. L.; Rothe, M.; Röckmann, T. Vehicle emissions of greenhouse gases and related tracers from a tunnel study: $CO:CO_2$, $N_2O:CO_2$, $CH_4:CO_2$, $O_2$ $:CO_2$ ratios, and the stable isotopes $^{13}C$ and $^{18}O$ in $CO_2$ and CO. *Atmos. Chem. Phys.* **2014**, *14*, 2105–2123.

3. Naus, S.; Röckmann, T.; Popa, M. The isotopic composition of CO in vehicle exhaust. *Atmos Environ*. **2018**, *177*, 132–142.

4. Andres, R. J.; Marland, G.; Boden, T.; Bischof, S. Carbon Dioxide Emissions from Fossil Fuel Consumption and Cement Manufacture, 1751–1991; and an Estimate of Their Isotopic Composition and Latitudinal Distribution. Technical report. Oak Ridge National Lab., TN (United States); Oak Ridge Inst. for Science and Education, TN (United States) **1994**.

5. Horvath, B.; Hofmann, M. E. G.; Pack, A. On the triple oxygen isotope composition of carbon dioxide from some combustion processes. *Geochem. Cosmochim. Acta.* **2012**, *95*, 160–168.

6. Röckmann, T.; Brenninkmeijer, C. A. M.; Saueressig, G.; Bergamaschi, P.; Crowley, J. N.; Fischer, H.; Crutzen, P. J. Mass independent fractionation of oxygen isotopes in atmospheric CO due to the reaction CO + OH. *Science.* **1998,** *281*, 544-546.

7. Huff, A. K.; Thiemens, M. H. $^{17}O/^{16}O$ and $^{18}O/^{16}O$ isotope measurements of atmospheric carbon monoxide and its sources. *Geophys. Res. Lett.* **1998**, *25*, 3509–3512.

8. Kato, S.; Akimoto, H.; Bräunlich, M.; Röckmann, T.; Brenninkmeijer, C. A. M. Measurements of stable carbon and oxygen isotopic compositions of CO in automobile exhausts and ambient air from semi-urban Mainz, Germany. *Geochem. J*. **1999**, *33*, 73–77.

9. Vimont, I. J.; Turnbull, J. C.; Petrenko, V. V.; Place, P. F.; Sweeney, C.; Miles, N.; Richardson, S.; Vaughn, B. H.; White, J. W. C. An improved estimate for the $\delta^{13}C$ and $\delta^{18}O$ signatures of carbon monoxide produced from atmospheric oxidation of volatile organic compounds. *Atmos. Chem. Phys.* **2019**, *19*, 8547–8562.

10. Pandey, A.; Venkataraman, C. Estimating emissions from the Indian transport sector with on-road fleet composition and traffic volume. *Atmos. Environ.* **2014**, *98*, 123-133.

11. Baidya, S.; Borken-Kleefeld, J. Atmospheric emissions from road transportation in India. *Energy Policy.* **2009**, *37*, 3812-3822.

12. GTZ, Sustainable transport: a sourcebook for policy-makers in developing cities. 2012. Division 44, module 4c.

13. Yan, F.; Winijkula, E.; Junga, S.; Bonda, T. C.; Streets, D. G. Global emission projections of particulate matter (PM): I. Exhaust emissions from on-road vehicles. *Atmos. Environ.* **2011**, *45*, 4830-4844.

14. Vimont, I. J.; Turnbull, J. C.; Petrenko, V. V.; Place, P. F.; Karion, A.; Miles, N. L.; Richardson, S. J.; Gurney, K.; Patarasuk, R.; Sweeney, C.; Vaughn, B.; White, J. W. C. Carbon monoxide isotopic measurements in Indianapolis constrain urban source isotopic signatures and support mobile fossil fuel emissions as the dominant wintertime CO source. *Elem Sci. Anth.* **2017,** *5*, 63.

15. Dickerson, R. R.; Andreae, M. O.; Campos, T.; Mayol-Bracero, O. L.; Neusuess, C.; Streets, D. G. Analysis of black carbon and carbon monoxide observed over the Indian Ocean: Implications for emissions and photochemistry. *J. Geophys. Res.* **2002**, *107*, 8017.

16. O'Leary, M. H. Carbon isotopes in photosynthesis. *Bioscience.* **1998**, *38*, 328–336.

17. Qiu, C. The isotopic signatures of CO in biomass burning emission. *Master Thesis IMAU, Utrecht University.* **2019**.

18. Kato, S.; Akimoto, H.; Rockmann, T.; Braunlich, M.; Brenninkmeijer, C. A. M. Stable isotopic compositions of carbon monoxide from biomass burning experiments. *Atmos. Environ.* **1999**, *33*, 4357–4362.

19. Röckmann, T.; Jöckel, P.; Gros, V.; Bräunlich, M.; Possnert, G.; Brenninkmeijer, C. A. M. Using $^{14}C$, $^{13}C$, $^{18}O$ and $^{17}O$ isotopic variations to provide insights into the high northern latitude surface CO inventory. *Atmos. Chem. Phys.* **2002**, *2*, 147–159.

20. Paliwal, U.; Sharma, M.; Burkhart, J. F. Monthly and spatially resolved black carbon emission inventory of India: uncertainty analysis. *Atmos. Chem. Phys.* **2016**, *16*, 12457–12476.

21. Keeling, C. D. The concentration and isotopic abundances of atmospheric carbon dioxide in rural areas, *Geochim. Cosmochim. Acta.* **1958**, *13*, 322–334.

22. Lobert, J. M.; Harris, J. M. Trace gases and air mass origin at Kaashidhoo, Indian Ocean. *J. Geophys. Res.* **2002**, *107*, 8013.

23. Mak, J. E.; Kra, G.; Sandomenico, T.; Bergamaschi, P. The seasonally varying isotopic composition of the sources of carbon monoxide at Barbados, West Indies. *J. Geophys. Res.* **2003**, *108*, 4635.

24. Stevens, C. M.; Wagner, A. F. The role of isotope fractionation effects in atmospheric chemsitry, *Z. Naturforsch. A.* **1989**, *44*, 376–384.

25. Andersson, A.; Deng, J.; Ke, D.; Zheng, M.; Yan, C.; Sköld, M.; Gustafsson, Ö. Regionally-varying combustion sources of the January 2013 severe haze events over eastern China. *Environ. Sci. Technol.* **2015**, *49*, 2038–4496.

26. Winiger, P.; Andersson, A.; Eckhardt, S.; Stohl, A.; Semiletov, I. P.; Dudarev, O. V.; Charkin, A.; Shakova, N.; Klimont, Z.; Heyes, C.; Gustafsson, Ö. Siberian Arctic black carbon sources constrained by model and observation. *Proc. Natl. Acad. Sci. USA.* **2017**, *114*, 1054–1061.

27. DeMore, W. B.; Sander, S. P.; Golden, D. M.; Hampson, R. F.; Kurylo, M. L.; Howard, C. J.; Ravishankara, A. R.; Kolb, C. E.; Molina, M. J. Chemical kinetics and photochemical data for use in stratospheric modeling. *JPL Publication.* **1997**, *12*, 97-4.

28. Fulle, D.; Hamann, H. F.; Hippler, H.; Troe, J. High-pressure range of addition reactions of HO. II. Temperature and pressure dependence of the reaction HO + CO → HOCO → CO2. *J. Chem. Phys.* **1996**, *105*, 983–1000.

29. Lelieveld, J.; Gromov, S.; Pozzer, A.; Taraborrelli, D. Global tropospheric hydroxyl distribution, budget and reactivity. *Atmos. Chem. Phys.* **2016**, *16*, 12477–12493.

30. Nisbet, E. G.; Manning, M. R.; Dlugokencky, E. J.; Fisher, R. E.; Lowry, D.; Michel, S. E.; Myhre, C. L.; Platt, S. M.; Allen, G.; Bousquet, P.; Brownlow, R.; Cain, M.; France, J. L.; Hermansen, O.; Hossaini, R.; Jones, A. E.; Levin, I.; Manning, A. C.; Myhre, G.; Pyle, J. A.; Vaughn, B. H.; Warwick, N. J.; White, J. W. C. Very strong atmospheric methane growth in the 4 years 2014–2017: Implications for the Paris Agreement. *Global Biogeochemical Cycles* **2019**, *33*, 318–342.

31. Bergamaschi, P.; Hein, R.; Heimann, M.; Crutzen, P. J. Inverse modeling of the global CO cycle 1. Inversion of CO mixing ratios. *J. Geophys. Res.* **2000,** *105*, 1909–1927.

32. Bergamaschi, P.; Hein, R.; Brenninkmeijer, C. A. M.; Crutzen, P. J. Inverse modeling of the global CO cycle: 2. Inversion of $^{13}C/^{12}C$ and $^{18}O/^{16}O$ isotope ratios. *J. Geophys. Res.* **2000**, *105*, 1929–1945.

33. Holloway, T.; Levy II, H.; Kasibhatla, P. Global distribution of carbon monoxide. *J. Geophys. Res.* **2000**, *105*, 12123–12147.

34. Kasibhatla, P. S.; Arellano, A.; Logan, J. A.; Palmer, P. I.; Novelli, P. Top-down estimate of a large source of atmospheric carbon monoxide associated with fuel combustion in Asia. *Geophys. Res. Lett.* **2002**, *29*, 1900.

35. Arellano, A. F.; Kasibhatla, P. S.; Giglio, L.; van der Werf, G. R.; Randerson, J. T. Top-down estimates of global CO sources using MOPITT measurements. *Geophys. Res. Lett.* **2004**, *31*, L01104.

36. Müller, J. F.; Stavrakou, T. Inversion of CO and NOx emissions using the adjoint of the images model. *Atmos. Chem. Phys.* **2005**, *5*, 1157–1186.

37. Arellano, A. F.; Kasibhatla, P. S.; Giglio, L.; van der Werf, G. R.; Randerson, J. T.; Collatz, G. J. Time-dependent inversion estimates of global biomass-burning CO emissions using measurement of pollution in the troposphere (MOPITT) measurements. *J. Geophys. Res.* **2006**, *111*, D09303.

38. Folberth, G. A.; Hauglustaine, D. A.; Lathière, J.; Brocheton, F. Interactive chemistry in the Laboratoire de Météorologie Dynamique general circulation model: model description and impact analysis of biogenic hydrocarbons on tropospheric chemistry. *Atmos. Chem. Phys*. **2006**, *6*, 2273–2319.

39. Duncan, B. N.; Logan, J. A.; Bey, I.; Megretskaia, I. A.; Yantosca, R. M.; Novelli, P. C.; Jones, N. B.; Rinsland, C. P. Global budget of CO, 1988–1997: Source estimates and validation with a global model. *J. Geophys. Res. Atmos.* **2007**, *112*, D22301.

40. Park, K.; Emmons, L. K.; Wang, Z.; Mak, J. E. Joint application of concentration and $\delta^{18}O$ to investigate the global atmospheric CO budget. *Atmosphere.* **2015**, *6*, 547–578.

41. Zheng, B.; Chevallier, F.; Yin, Y.; Ciais, P.; Fortems-Cheiney, A.; Deeter, M. N.; Parker, R. J.; Wang, Y.; Worden, H. M.; Zhao, Y. Global atmospheric carbon monoxide budget 2000–2017 inferred from multi-species atmospheric inversions. *Earth Syst. Sci. Data.* **2019**, *11*, 1411–1436.

42. Stevens, C. M.; Krout, L.; Walling, D.; Venters, A. The Isotopic Composition of Atmospheric Carbon Monoxide. *Earth Planet. Sc. Lett.* **1972**, *16*, 147–165.

43. Saurer, M.; Prévôt, A. S. H.; Dommen, J.; Sandradewi, J.; Baltensperger, U.; Siegwolf, R. T. W. The influence of traffic and wood combustion on the stable isotopic composition of carbon monoxide. *Atmos. Chem. Phys.* **2009**, *9*, 3147-3161.

44. Brenninkmeijer, C. A. M.; Röckmann, T.; Braunlich, M.; Jockel, P.; Bergamaschi, P. Review of Progress in Isotope Studies of Atmospheric Carbon Monoxide. *Chemosphere- Global Change Science*. **1999**, *1*, 33-52.

45. Brenninkmeijer, C. A. M.; Röckmann, T. Principal factors determining the $^{18}O/^{16}O$ ratio of atmospheric CO as derived from observations in the southern hemispheric troposphere and lowermost stratosphere. *J. Geophys. Res.* **1997**, *102*, 25477.

46. Cantrell, C. A.; Shetter, R. E.; McDaniel, A. H.; Calvert, J. G.; Davidson, A.; Lowe, D. C.; Tyler Ralph, S. C.; Cicerone, J.; Greenberg, J. P. Carbon kinetic isotope effect in the oxidation of methane by hydroxyl radicals. *J. Geophys. Res.* **1990**, *95*, 22455-22462.

47. Kurokawa, J.; Ohara, T.; Morikawa, T.; Hanayama, S.; Janssens-Maenhout, G.; Fukui, T.; Kawashima, K.; Akimoto, H. Emissions of air pollutants and greenhouse gases over Asian regions during 2000–2008: Regional Emission inventory in ASia (REAS) version 2. *Atmos. Chem. Phys.* **2013**, *13*, 11019–11058.

48. Jain, N.; Bhatia, A.; Pathak, H. Emission of air pollutants from crop residue burning in India. *Aerosol Air Qual. Res.* **2014**, *14*, 422−430.

49. Kurokawa, J.; Ohara, T. Long-term historical trends in air pollutant emissions in Asia: Regional Emission inventory in ASia (REAS) version 3. *Atmos. Chem. Phys.* **2020**, *20*, 12761–12793.

50. Metropolis, N.; Rosenbluth, A. W.; Rosenbluth, M. N.; Teller, A. H.; Teller, E. Equation of state calculations by fast computing machines. *J. Chem. Phys.* **1953**, *21*, 1087-1092.

51. Hastings, W. K. Monte Carlo sampling methods using Markov chains and their applications. *Biometrika.* **1970**, *57*, 97-109.
